# Small Molecule Decoys of Aggregation for Elimination of Aβ-Peptide Toxicity

**DOI:** 10.1021/acschemneuro.2c00649

**Published:** 2023-04-14

**Authors:** Sho Oasa, Valentina L. Kouznetsova, Ann Tiiman, Vladana Vukojević, Igor F. Tsigelny, Lars Terenius

**Affiliations:** † Department of Clinical Neuroscience, Center for Molecular Medicine, Karolinska Institutet, SE-171 76 Stockholm, Sweden; ‡ San Diego Supercomputer Center, University of California San Diego, La Jolla, California 92093-0505, United States; § Department of Neurosciences, University of California San Diego, La Jolla, California 92093-0819, United States; ∥ Department of Clinical Neuroscience, Karolinska University Hospital, Karolinska Institutet, SE-17176 Stockholm, Sweden

**Keywords:** Alzheimer’s disease, Aβ aggregation, inhibitor, molecular design, experimental therapy

## Abstract

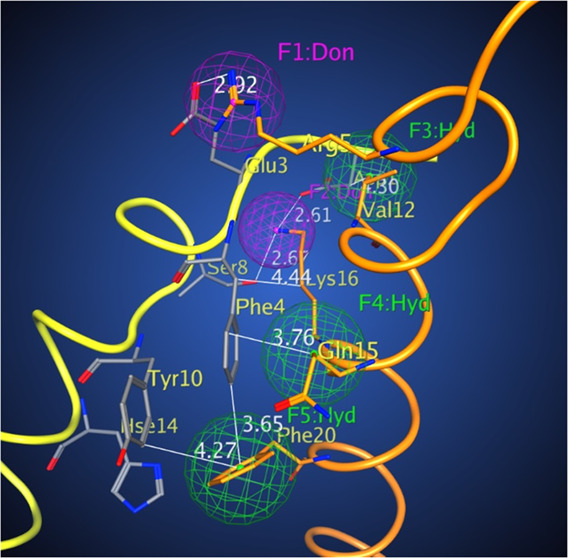

Several lines of evidence suggest that a characteristic of the neuropathology of Alzheimer’s disease (AD) is the aggregation of the amyloid beta peptides (Aβ), fragments of the human amyloid precursor protein (hAPP). The dominating species are the Aβ40 and Aβ42 fragments with 40 and 42 amino acids, respectively. Aβ initially forms soluble oligomers that continue to expand to protofibrils, suggestively the neurotoxic intermediates, and thereafter turn into insoluble fibrils that are markers of the disease. Using the powerful tool of pharmacophore simulation, we selected small molecules not known to possess central nervous system (CNS) activity but that might interact with Aβ aggregation, from the NCI Chemotherapeutic Agents Repository, Bethesda, MD. We assessed the activity of these compounds on Aβ aggregation using the thioflavin T fluorescence correlation spectroscopy (ThT-FCS) assay. Förster resonance energy transfer-based fluorescence correlation spectroscopy (FRET-FCS) was used to characterize the dose-dependent activity of selected compounds at an early stage of Aβ aggregation. Transmission electron microscopy (TEM) confirmed that the interfering substances block fibril formation and identified the macrostructures of Aβ aggregates formed in their presence. We first found three compounds generating protofibrils with branching and budding never observed in the control. One compound generated a two-dimensional sheet structure and another generated a double-stranded filament. Importantly, these compounds generating protofibrils with altered macrostructure protected against Aβ-induced toxicity in a cell model while showing no toxicity in a model of cognition in normal mice. The data suggest that the active compounds act as decoys turning the aggregation into nontoxic trajectories and pointing toward novel approaches to therapy.

## Introduction

There is strong evidence that a key process in the neuropathology of Alzheimer’s disease (AD) is the aggregation of amyloid beta peptide (Aβ). The dominating species are 40–42 amino acids long, Aβ_40_ and Aβ_42_ generated by enzymatic cleavage of the amyloid precursor protein (APP). The very nature of enzymatic process is unusual in that the Aβ peptides easily form a dimer, which then continues to expand in either a linear fashion, generating protofibrils and, thereafter, fibrils as end products, or into a circular fashion, generating ring-shaped aggregates. The fibrils serve as histological markers, but toxic activity appears due to shorter soluble aggregates. Due to difficulties in “freezing” the process at any defined step, it is still unclear which species are toxic. In fact, different morphological structures may possess different kinds of toxicities.^1^ Attempts to neutralize all potentially toxic species by immunoadsorption, or select species that seem to be the most toxic, have largely failed.^2^ The amyloid hypothesis^3^ has been questioned as a target for therapeutic intervention in AD, mostly due to therapeutic failures. A recent commentary attributes this paradox by proposing that the disease process primarily affects the endocytosis of APP, tau, and other proteins. The degenerative process is constrained in a “hub,” which irradiates through different lines (“spokes”). The consequence is the increased degradation of APP and the increased formation of Aβ and phosphorylation of tau.^4^ In contrast to the common assumption of a linear model, this model suggests a parallel development of pathological processes.

Another line of research has opened for the existence of an expansion of the number of APP genes (gene copy number variation) in the AD brain. The gene expansion generates a complex gene transcription pattern and different transcripts with or without the Aβ coding sequence.^5^

In an early study,^6^ we took advantage of fluorescence correlation spectroscopy (FCS) to follow without interference aggregation of Aβ in real time in the test tube. We defined a segment, KLVFF, Aβ (16–20), as the minimum sequence for aggregation and demonstrated that this segment could be targeted for drug development. The simple addition of a proline-rich peptide to break β-sheets increased affinity.^7^ A larger fragment Aβ (13–26) “clamped” to stabilize the structure was found to protect against neurotoxicity in hippocampal slices.^8^

Tsigelny and co-workers successfully identified and developed small molecule drug candidates using the strategies of pharmacophore-based virtual screening for ligand-based pharmacophores^9,10^ and interface-based pharmacophores.^10^

Molecular dynamics (MD) simulations and pharmacophore-based docking of the MD conformers revealed that the Aβ_42_ dimerization interface could be a proper point for drug intervention.^11^ Based on the hypothesis that small molecules, which are able to fit this interface would selectively inhibit Aβ_42_ dimerization and thereby prevent expansion to the toxic oligomeric states, we developed a pharmacophore model based on interacting residues of Aβ_42_ monomers and successfully identified potential drug-lead candidates inhibiting Aβ_42_ and, as we show in our experiments below, also Aβ_40_ oligomerization. The inhibitory effect of candidate compounds was characterized *in vitro* using several techniques: (1) the FCS measurement of thioflavin T (ThT) fluorescence when interacting with peptide aggregates in a β-sheet secondary structure,^12^ (2) transmission electron microscopy (TEM) of precipitates, and (3) FCS integrated with Förster resonance energy transfer (FRET-FCS)^13^ to assess early aggregation states and characterize the dose–response relationships. Compounds found to be active in Aβ aggregation reduced Aβ-mediated toxicity in cell culture and showed no toxicity in a behavioral mouse model for testing cognitive abilities. These data may provide the basis for the elucidation of novel small molecule inhibitors of Aβ dimerization and lead to the development of novel AD therapeutics.

## Results

**Figure 1:**
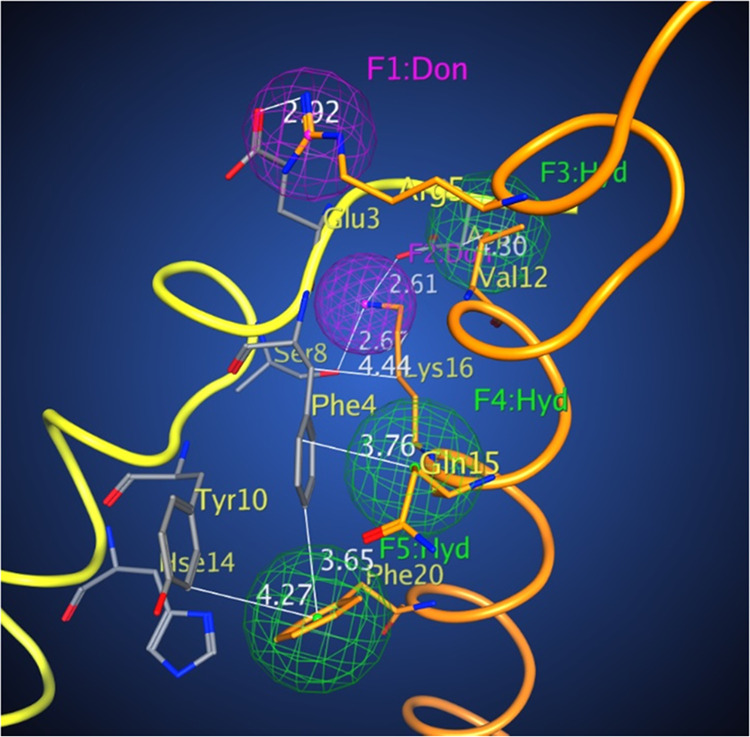
Aβ_42_ dimer interface used for the development of the pharmacophore model (for details, see the Supporting Information). Color scheme: orange residues: Aβ_42_ monomer 1; yellow with gray residues: Aβ_42_ monomer 2; red: oxygen; blue: nitrogen, magenta: donors, and green: hydrophobic centers.

MD simulations determined the most stable conformers of Aβ_42_, and docking analysis showed that these conformers formed Aβ_42_ dimers at the membrane surface. Consecutive docking of Aβ_42_ monomers to the homodimer revealed that an annular heptamer is being formed (Figure S1). Importantly, the Aβ_42_ dimerization surface included a set of very specific polar and hydrophobic interactions (Figure 1 and Table S1).

Based on these simulation data, we applied pharmacophore-based docking to the Aβ_42_ dimerization surface to identify molecules that hold the potential to interfere with Aβ_42_ dimer and oligomer formation. In particular, we have analyzed the possible intermolecular contacts between the neighboring Aβ_42_ monomers (Figure 1 and Table S1) to develop a pharmacophore hypothesis comprising complementary polar and hydrophobic features. To identify scaffolds able to disrupt the Aβ_42_ dimer interface, we considered one Aβ_42_ monomer as a receptor and the other as a ligand. A schematic drawing of the Aβ_42_ homodimerization interface is shown in Figure S2. In Figures 1 and S2, the monomer receptor (M1) and its residues are colored orange, while the monomer ligand (M2) is yellow, and its residues are gray. The pharmacophore is developed on the base of the monomer receptor (M1). One can see that the Aβ_42_ dimer interface has a unique profile and is amenable to the design of selective pharmacophore centers having two conserved positive residues—Arg 5 and Lys 16 of the monomer receptor (M1) that interact with Glu 3 and Asp 7, and Ser 8 of the monomer ligand (M2), correspondingly, forming two donor centers (Figure 1, dark magenta). There are also three hydrophobic centers—Val 12, the hydrophobic stem of Gln 15 that interacts with Asp 7 and Phe 4, and Phe 20 interacting with Phe 4 and Tyr 10 (Figure 1, green).

Based on these data, a complementary combination of residues could be considered for pharmacophore development (Figure S2). Computational docking of compounds using the pharmacophore centers found in the Aβ_42_ dimerization surface (Figure 1), identified in the NCI Open Compound Repository^a^ over 30 candidate molecules with the potential to block 4–5 centers. Visual inspection of molecular geometry identified some common characteristics, notably two aromatic nuclei with a rigid linker and a flat overall structure, and 8 compounds were selected. In a second run, additional 7 compounds were selected, including one with an analogous structure, and the others not (Figure S3).

**Figure 2:**
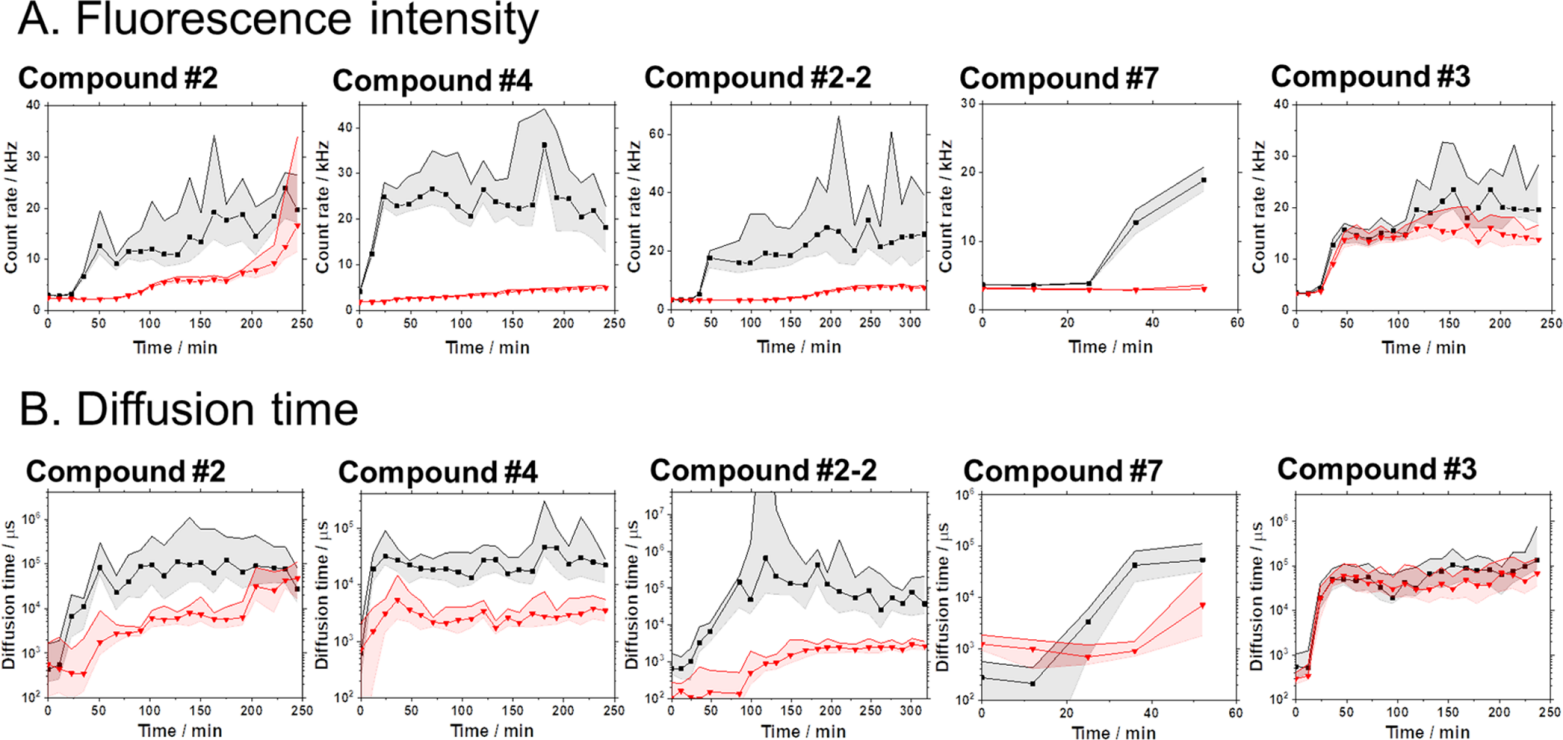
Effects of test compounds on Aβ_40_ aggregation visualized by thioflavin T fluorescence correlation spectroscopy (ThT-FCS). Time-lapse FCS measurements determined ThT fluorescence intensity and diffusion time, allowing estimation of the amount and size of ThT-positive Aβ_40_ aggregates at each time point. (A) Median of ThT fluorescence intensity in 30 × 10 s FCS measurements. (B) Median of the diffusion time of ThT-positive Aβ_40_ aggregates determined in 30 × 10 s FCS measurements. Black: the control experiment with no test compound, red: measurement with the test compound with an equimolar concentration (10 μM) of the test compound and monomeric Aβ_40_ peptide. The shaded region shows the 25–75 quartile range of values determined in 30 FCS measurement repeats.

We then used ThT-FCS^12^ as a screening test (Figures 2 and S4) since ThT gains fluorescence when binding to Aβ_40_ or Aβ_42_ oligomers enriched in a β-sheet secondary structure. Based on the change in ThT fluorescence intensity over time (Figure 2A), we identified two basic actions of the tested compounds, no effect (#3) or apparent inhibition (#2, #4, #2-2, #7). Moreover, the size of Aβ_40_ aggregates, estimated from diffusion times, changed with inhibitory compounds (Figure 2B). In particular, compound #2 delayed the time to reach the same level of ThT fluorescence intensity and significantly decreased the diffusion time. The other compounds also effectively blocked the increase in ThT fluorescence intensity and significantly decreased the diffusion time. Confocal laser scanning microscopy (CLSM) fluorescence imaging revealed that large aggregates precipitate on the coverslip at the endpoint of the ThT-FCS time series (Figure S7). While compounds #4 and #2-2 clearly decreased the number of Aβ_40_ precipitates compared with no compound, compound #7 caused the formation of large and structurally distinct precipitates of Aβ_40_. These results suggested that compounds #2, #4, and #2-2 are potential inhibitors of Aβ_40_ aggregation to toxic intermediates and that compound #7 accelerated Aβ_40_ aggregation. Compounds #1 and #6-2 could not be tested using the ThT-FCS assay due to high autofluorescence and self-assembly in the buffer. They were deemed unsuitable for further analysis. Further details are given in Figures S5 and S6.

**Figure 3:**
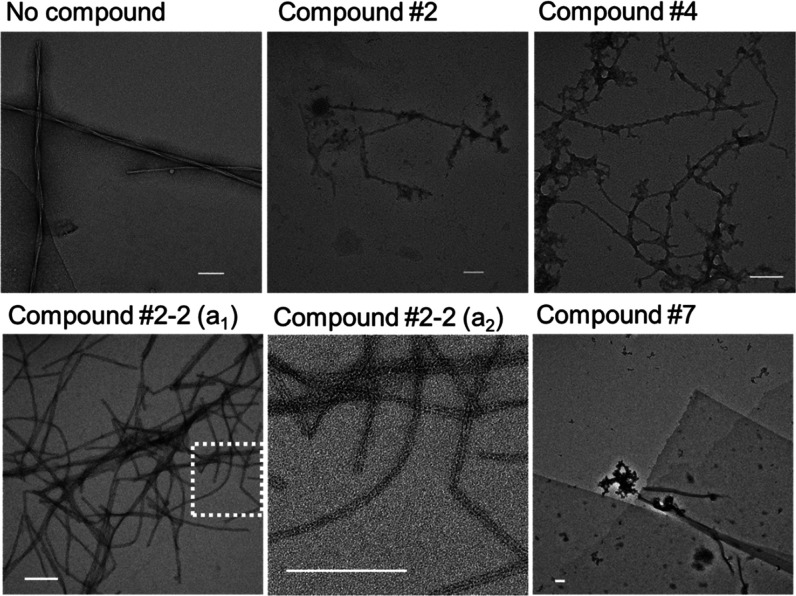
Transmission electron microscopy (TEM) images of Aβ_40_ aggregates formed in a 10 μM solution of Aβ_40_ alone (no compound) or with an equimolar concentration of test compounds #2, #4, #2-2 (a_1_), or #7. (a_2_) Enlarged image of the region in the white-dotted inset shown in panel (a_1_). Scale bar: 100 nm.

To address the structural differences of Aβ_40_ precipitates (Figure S7), we used TEM to visualize Aβ_40_ aggregates formed in the presence of compounds #2, #4, #5, #2-2, and #7 (Figure 3). TEM confirmed the presence of precipitates. Intriguingly, the macroanatomy of the precipitates differed. Compounds #2 and #4 produced protofibrils which were distorted with buds and branches never observed in the control. Compound #2-2 gave evidence for a uniquely double-stranded unbranched protofibrillar morphology in spaghetti-like agglomerates. Compound #7 led to the generation of Aβ_40_ aggregates that acquired a sheet structure, with no evidence for either protofibrillar or fibrillar structures (Figures 3 and S8). The sheets were of a very regular rectangular shape, apparently monolayer. Certain sheets had sharp edges and 90° corners (Figure 3). We further characterized the thickness of protofibrillar/fibrillar structures observed in TEM images (Figure S9). Native Aβ_40_ protofibrils were 8.0 ± 0.8 nm thick and mature fibrils were 15 ± 2.8 nm thick. In the presence of compounds #2, #4, and #5, Aβ_40_ formed protofibrils of similar thickness as Aβ_40_ alone. Interestingly, compound #2-2 generated double-stranded protofilament, with each strand being significantly thinner, 3.6 ± 0.8 nm. These results suggest that compounds #2, #4, and #2-2 interfere with fibrillar structure formation and that compound #2-2 generates double-stranded filaments *via* a different assembling trajectory.

ThT-FCS and TEM do not characterize the effect of test compounds on Aβ peptide association in the early stage of the aggregation processes. Fluorescence cross-correlation spectroscopy (FCCS), which is effective in quantifying protein–protein interactions,^14^ could, however, not be applied since cross-correlation was not observed in a mixture of fluorescently labeled peptides, HiLyte Fluor 488-Aβ (Aβ_40,488_) and HiLyte Fluor 647-Aβ (Aβ_40,647_), due to large excess of unbound Aβ_40,488_ and Aβ_40,647_ in the mixture (Figure S10).

**Figure 4:**
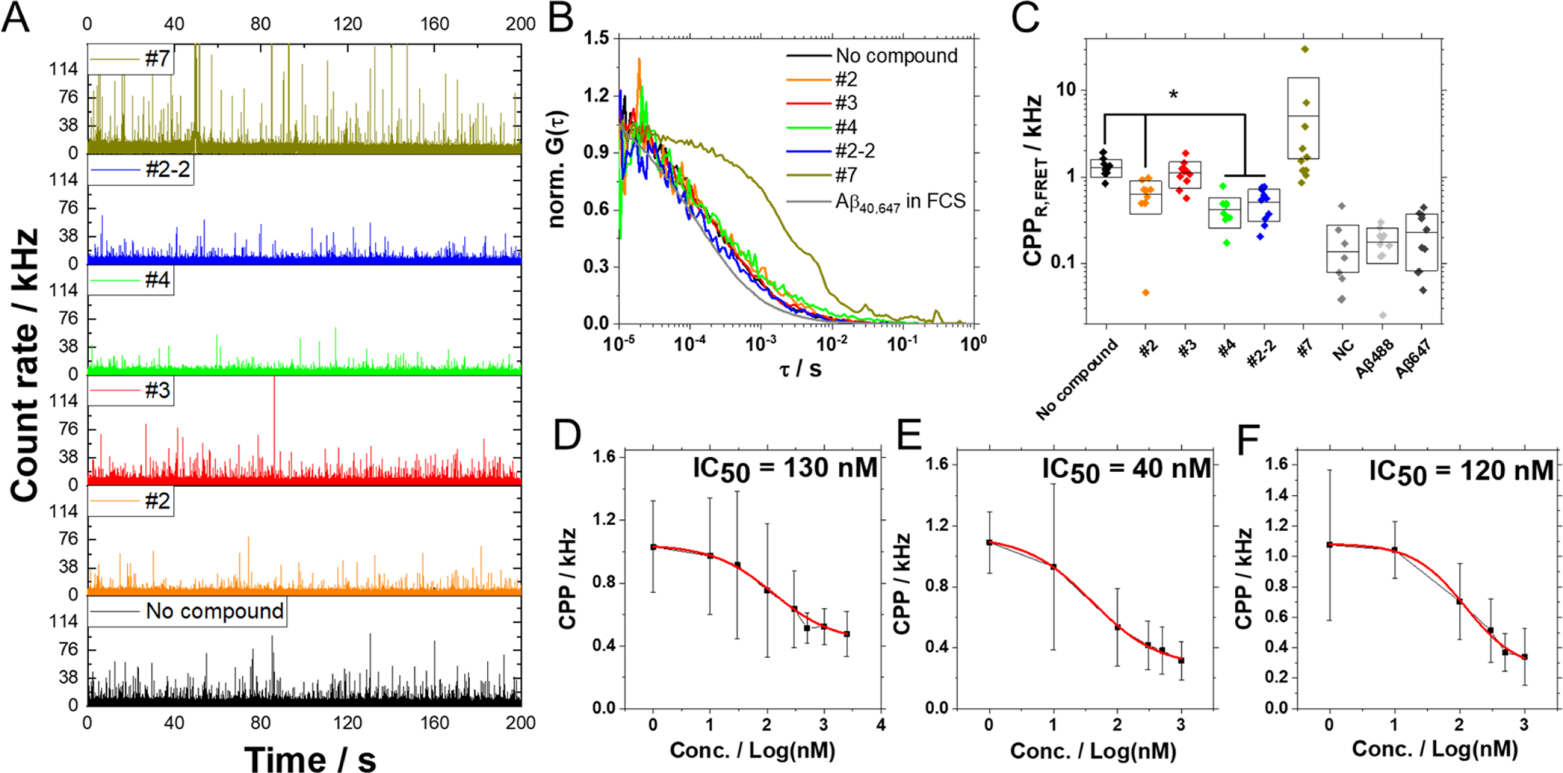
Characterization of test compound effects on early stages of Aβ_40_ aggregation by FRET-FCS. (A) Fluorescence intensity fluctuations originating through FRET. FRET-FCS measurements in solutions of the fluorescently labeled Aβ_40_ peptide without/with the test compound. The concentration of reactants: 100 nM HiLyte 488-Aβ_40_ (Aβ_40,488_), 200 nM HiLyte647-Aβ_40_ (Aβ_40,647_), and 300 nM test compound. (B) Corresponding autocorrelation curves normalized to the same amplitude, *G*(τ) = 1 at τ = 10 μs. The longer the decay time of the autocorrelation curve, the larger the size of the FRET-positive Aβ_40_ aggregates. Gray: the autocorrelation curve acquired in a solution of Aβ_40,647_ using conventional single-color FCS measurements in the red channel. (C) Molecular brightness, reflected by counts per particle (CPP); expressed as the average ± standard deviations: no compound, 1.3 ± 0.3; #2, 0.6 ± 0.3; #3, 1.1 ± 0.4; #4, 0.4 ± 0.2; #2-2, 0.5 ± 0.2; #7, 5.1 ± 9.0. Statistical analysis was performed against no compound (*p < 0.001). (D–F) Dose–response curves showing the effect of compounds #2 (D), #4 (E), and #2-2 (F) on Aβ_40_ aggregation.

We, therefore, decided to use FCS integrated with FRET (FRET-FCS) to characterize compound effects at an early stage of the Aβ aggregation process using fluorescently labeled Aβ_40,488_ and Aβ_40,647_ (Figure S11).^13^ Fluorescence intensity traces clearly showed a reduction of fluorescence bursts with compounds #4 and #2-2 and an enhancement with compound #7 (Figure 4A). The FRET-FCS autocorrelation curves showed a longer correlation time compared with normal FCS autocorrelation curves for Aβ_40,647_ (Figure 4B, gray)—the diffusion time measured in FRET-FCS experiments was around 300 μs, which is twice longer than the value obtained in standard FCS recording, 150 ± 10 μs (Figure S12), suggesting the presence of small oligomers consisting of 8, at most, monomer units. Compound #7 generated larger Aβ_40_ aggregates, 2 ± 2 ms, acting as an inducer of Aβ_40_ aggregation.

To assess compound effects on oligomer formation at the early stage, molecular brightness, assessed *via* counts per particle (CPP), was also examined (Figure 4C). Compounds #2, #4, and #2-2 significantly reduced the brightness of the FRET-positive molecule, suggesting that these compounds are effective in inhibiting Aβ oligomer formation at an early stage. Molecular brightness was reduced in a dose-dependent manner, with IC_50_ values of 130, 40, and 120 nM for compounds #2, #4, and #2-2, respectively (Figure 4D–F).

FRET-FCS also showed that Aβ_42_ oligomers are of comparable size to Aβ_40_ oligomers. We observed similar activity on aggregation by compounds #2, #4, and #2-2, while compound #7 increased molecular brightness and caused a smaller change compared to its effect on the Aβ_40_ peptide, suggesting lower efficacy with the Aβ_42_ peptide (Figure S13). TEM also showed that compounds #7 and #2-2, respectively, exert similar effects on the structural morphology of Aβ_42_ precipitates as on Aβ_40_ (Figure S14).

**Figure 5:**
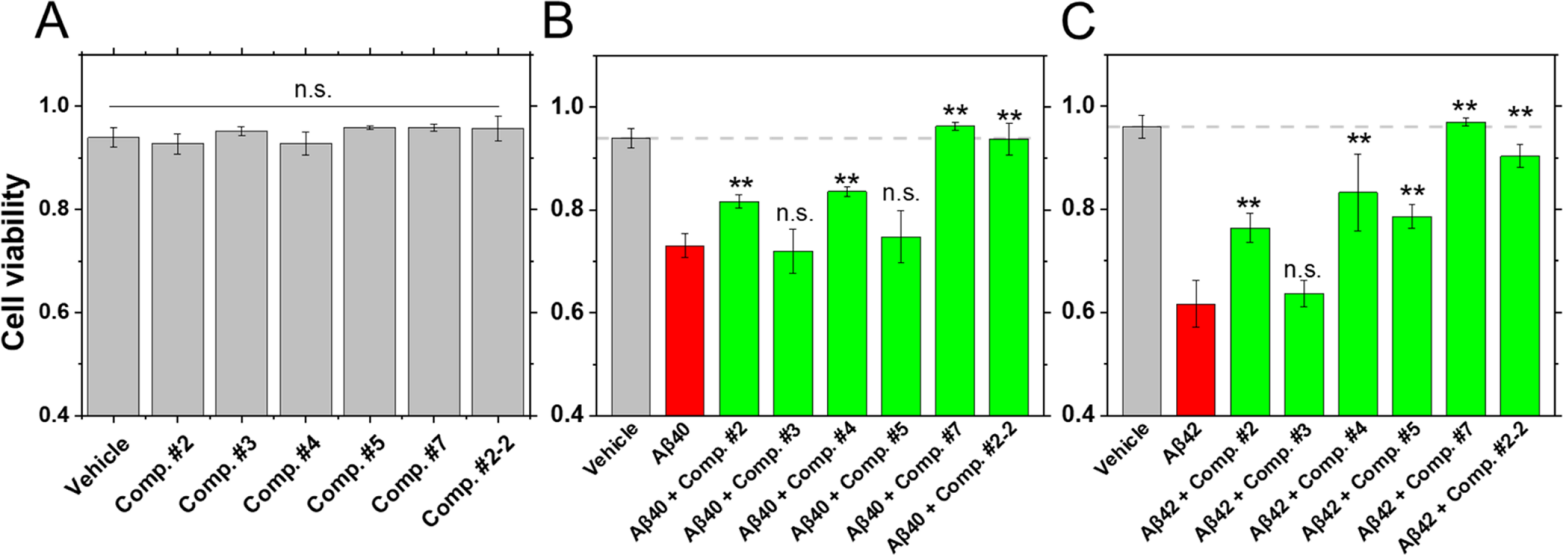
Impact of test compounds on Aβ-mediated cell toxicity. (A) Cell viability in the presence of test compounds alone. Test compound concentration was 3 μM in the vehicle (medium and HEPES buffer). (B) Cell viability in the presence of 3 μM Aβ_40_ and 3 μM compound. (C) Cell viability in the presence of 3 μM Aβ_42_ and compound. Gray: vehicle, red: 3 μM Aβ_40_ or Aβ_42_, and green: 3 μM Aβ_40_ or Aβ_42_ with 3 μM compounds. Average and standard deviation were calculated from three independent experiments. Statistical analysis was performed against the Aβ peptide alone (**p < 0.005).

We further tested the compound effect on Aβ aggregation-mediated cell toxicity. SH-SY5Y cells were incubated for 5 days with 3 μM compound dissolved in the medium and 4-(2-hydroxyethyl)-1-piperazineethanesulfonic acid (HEPES) buffer (vehicle, Figure 5A), with 3 μM Aβ_40_ + 3 μM compounds in the vehicle (Figure 5B) or with 3 μM Aβ_42_ + 3 μM compounds in the vehicle (Figure 5C). There were no significant differences in cell viability with compounds alone (Figure 5A), showing they are not toxic to the SH-SY5Y cells. Aβ_40_ and Aβ_42_ gave a reduction in cell viability compared to the vehicle (Figure 5B,C, gray bar). With compounds #2, #4, #7, and #2-2, cell viability recovered significantly with both Aβ peptides. On the other hand, compound #3 had no significant effect. Interestingly, compound #5 was also active against Aβ_42_-induced cell toxicity but not against Aβ_40_-induced cell toxicity. Active compounds were also tested for toxicity in a sensitive behavioral assay, the passive avoidance (PA) test in mice (Figure S15). No behavioral toxicity was recorded.

**Figure 6:**
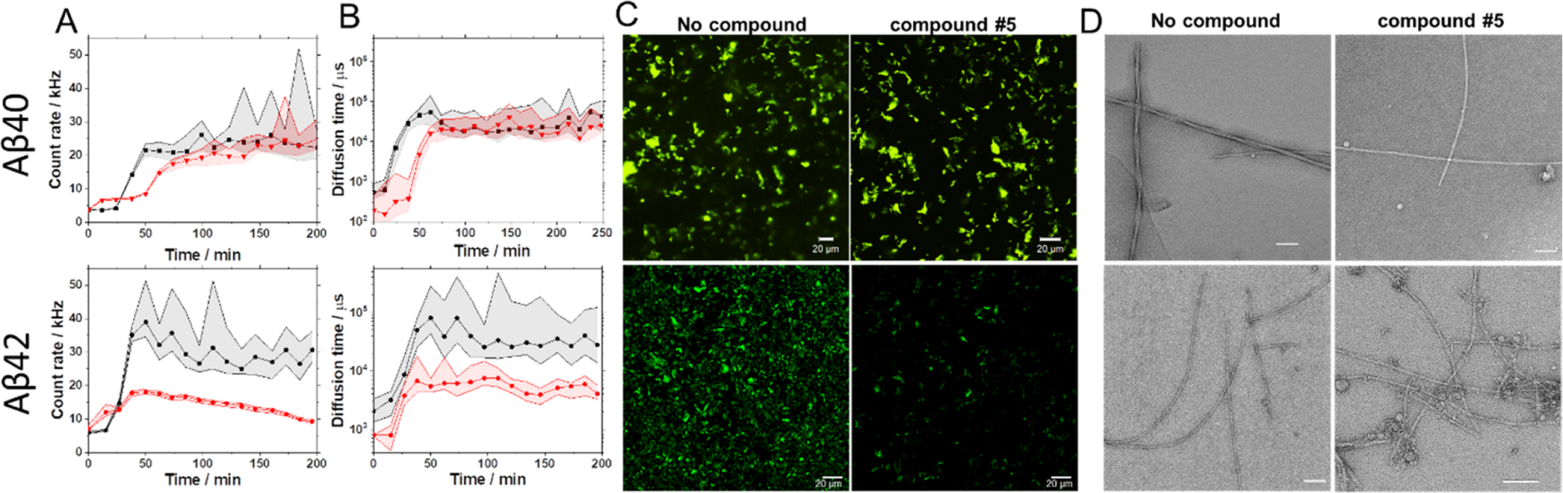
Comparative analysis of the effects of compound #5 on Aβ_40_ (upper panel) and Aβ_42_ (lower panel) aggregate formation visualized by ThT labeling and TEM. (A, B) Time-lapse FCS experiments showing changes in ThT fluorescence intensity (A) and diffusion time (B), *i.e.*, aggregate size over time. The median of ThT fluorescence intensity/diffusion time from 30 × 10 s FCS measurements is shown. The shaded region shows the 25–75 quartile range of values. Black: the control experiment with no test compound; red: experiments with test compound #5. The concentration of the Aβ peptide and test compound #5 are equimolar (10 μM). (C) Confocal images of ThT-positive Aβ_40_ and Aβ_42_ aggregates without/with #5 that precipitated on the cover glass. Scale bar: 20 μm. (D) TEM images of Aβ_40_ and Aβ_42_ aggregates formed without/with compound #5. Scale bar: 100 nm.

The apparent selectivity of compound #5 for Aβ_42_ aggregation prompted a more detailed analysis. Experiments with ThT labeling showed that compound #5 had differential effects on the fluorescence intensity time course (Figure 6A) and diffusion time of aggregates (Figure 6B), exhibiting a more potent effect on Aβ_42_ than Aβ_40_ aggregation. In addition, precipitation of large aggregates was only observed with Aβ_42_ but not with Aβ_40_ (Figure 6C). Using TEM, we could confirm that with compound #5, Aβ_42_ aggregates showed morphology earlier observed with compounds #2 and #4, protofibrils with buds and branches (Figure 6D).

## Discussion

It may seem an attractive way to interfere with Aβ aggregation as a therapeutic principle. Indeed, several studies in the past have been directed to search in compound libraries of molecules that interfere with Aβ aggregation and can serve as tool compounds toward potential therapeutics. With microarrays and a library of about 18,000 compounds, one new compound distinctly related to Pittsburgh compound-B was identified. Two compounds reduced the toxicity of Aβ_42_.^15^ A similar approach identified small molecules that protected against Aβ_42_ toxicity at micromolar concentration.^16^ Another study based on ion mobility spectrometry–mass-spectrometry (IMS–MS) showed that molecules that inhibit aggregation of the human islet amyloid polypeptide (hIAPP) and Aβ can be identified.^17^ The protocol allowed the identification of compound/monomer Aβ complexes, although three out of four positive compounds bound nonspecifically.^17^ This may be due to a limitation of the assay conditions, which would favor the detection of ion-pair complexes, whereas complexes based on hydrogen bonding only could be missed. This may be particularly worrisome since all of the hits we describe here suggest the importance of hydrogen bonding. Different possible strategies were recently reviewed.^18^ The current approach illustrates the potential of using molecular dynamics and docking of the dimerization interface. Further structural refinements would require expansion of the number of tested compounds. The common characteristic of an extended structure and a bridge connecting residues with an aromatic nucleus, and a planar surface may be significant. The selectivity of compound #5 for Aβ_42_ was unexpected and calls for further analysis. The large variation in macrostructure of aggregates is also a challenge.

The assembly of Aβ into amyloid aggregates is a highly ordered process, which is initiated in a stochastic manner by conformers of different shapes and sizes. To distinguish individual species is difficult and requires single-molecule techniques.^19^ The current data indicate that the process can be disturbed by small molecules with pharmacophores selected for interference at the dimer stage, with vast effects on the morphology of aggregates. Apparently, in the following step, the formation of fibrils is structurally more restrictive. Significantly, this may have relevance in studies with amyloid positron emission tomography (amyloid-PET), which records amyloid plaques that are primarily formed by amyloid fibrils.

Compound #5 is anomalous with selectivity for Aβ_42_ and turns aggregation into the macrostructure recorded with compounds #2 and #4 with both Aβ_40_ and Aβ_42_. Compound #5 is also chemically distinct from other tested compounds in having a single (large) pharmacophore. Potentially, this compound could be used to test the unique significance of Aβ_42_ deemed to be most relevant for toxicity.^3^

### Structural Features of Small Molecules Reducing Aβ Toxicity

**Figure 7:**
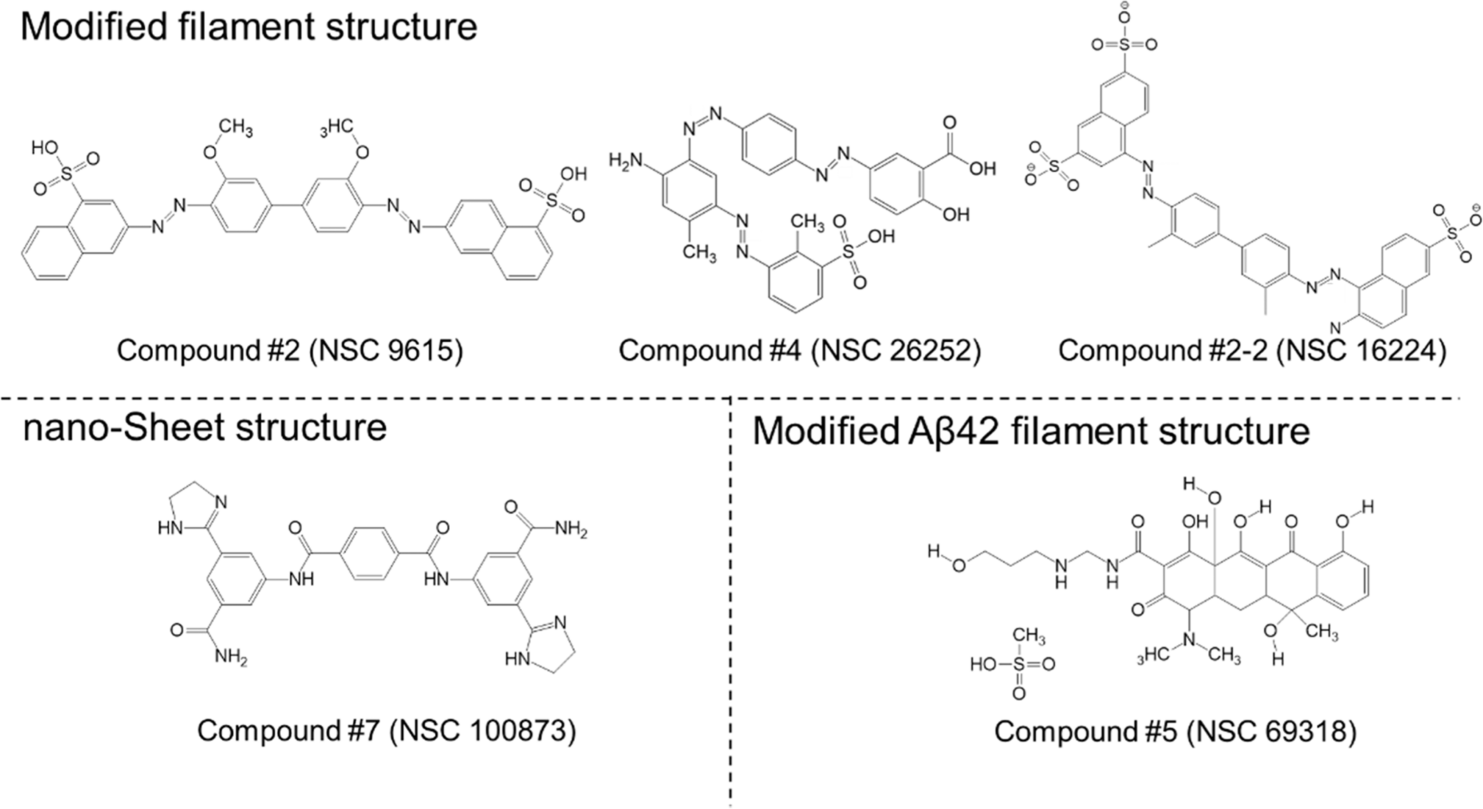
Structure of active compounds: #2 (NSC 9615), #4 (NSC26252), and #2-2 (NSC 16224) leading to the formation of the modified Aβ filament structure; #7 (NSC 100873) leading to Aβ nanosheet structure formation; #5 (NSC 69318) selectively modifying Aβ_42_ aggregation and filament structure formation only.

Using the pharmacophore search, over 30 compounds were identified. In the first round of experimental testing with eight compounds (Figure S3A), structural variation was emphasized, and several of the compounds were found to be active in the ThT-FCS prescreen. As already mentioned, two compounds could not be tested using this assay because of high autofluorescence and precipitation (see also Supporting Information Figures S5 and S6). Based on our initial experimental findings, it appeared that the active compounds #2, #4, #5, and #7 (Figure 7) fit four out of five pharmacophore features. We decided to test the model in a second round of experiments with 7 compounds (Figure S3B), of which only one, compound #2-2, fit pharmacophore’s five features of five and the others did not. In agreement with our hypothesis, only compound #2-2 was active.

The macrostructural changes that were apparently incompatible with fibrillation differed with the introduction of buds and branches (compounds #2 and #4), the formation of double-stranded filaments (compound #2-2), and the total absence of elongated, thread-like structures with the formation of a very regular, thin two-dimensional sheet structure (compound #7). Compounds #2-2 and #7 are particularly interesting since they completely block the toxicity of Aβ aggregates in cell culture (Figure 5). Strikingly, compound #2-2 is bifunctional with a covalent C–C linker between two identical aromatic moieties, whereas #7, which is symmetrical, consists of two identical moieties connected *via* a planar terephthalamide linker (Figure 7). The importance of this distinction has also been discussed in a recent review of bifunctional, proximity-inducing small molecules.^20^ It is interesting that in pathologic specimens, the amyloid fiber ultrastructure may vary, suggesting that also clinically, aggregation processes may follow slightly different pathways, for instance hiding the C-terminal (or N-terminal of Aβ).^21,22^ Such differences may, of course, have consequences for the therapeutic efficacy of antibodies generated against particular segments in defined conformations, which may not generally be accessible in immunotherapy.

The sheet structure induced by compound #7 has a precedent in KLVFFAK, an amyloid-forming fragment of the familial Italian form of AD.^23^ They observe that the nanosheet is very strong and backfolds. They conclude that this adds to the biotechnological use of amyloids and the emerging biotechnology field of amyloid aggregates. We notice by comparison that the initiation of sheet formation under our conditions is much more rapid than protofibril formation.

Compound #5 is a special case. It is chemically distinct from the other compounds and is only active against Aβ_42_, which is considered the most significant in the development of toxicity.^24^ The data clearly demonstrate that the C-terminal dipeptide sequence of Aβ_42_ is forcefully driving aggregation. This finding opens the potential for further studies of the relevant species for Aβ peptide toxicity.

### Structural Aspects of Pathologic Aβ Aggregation

**Figure 8:**
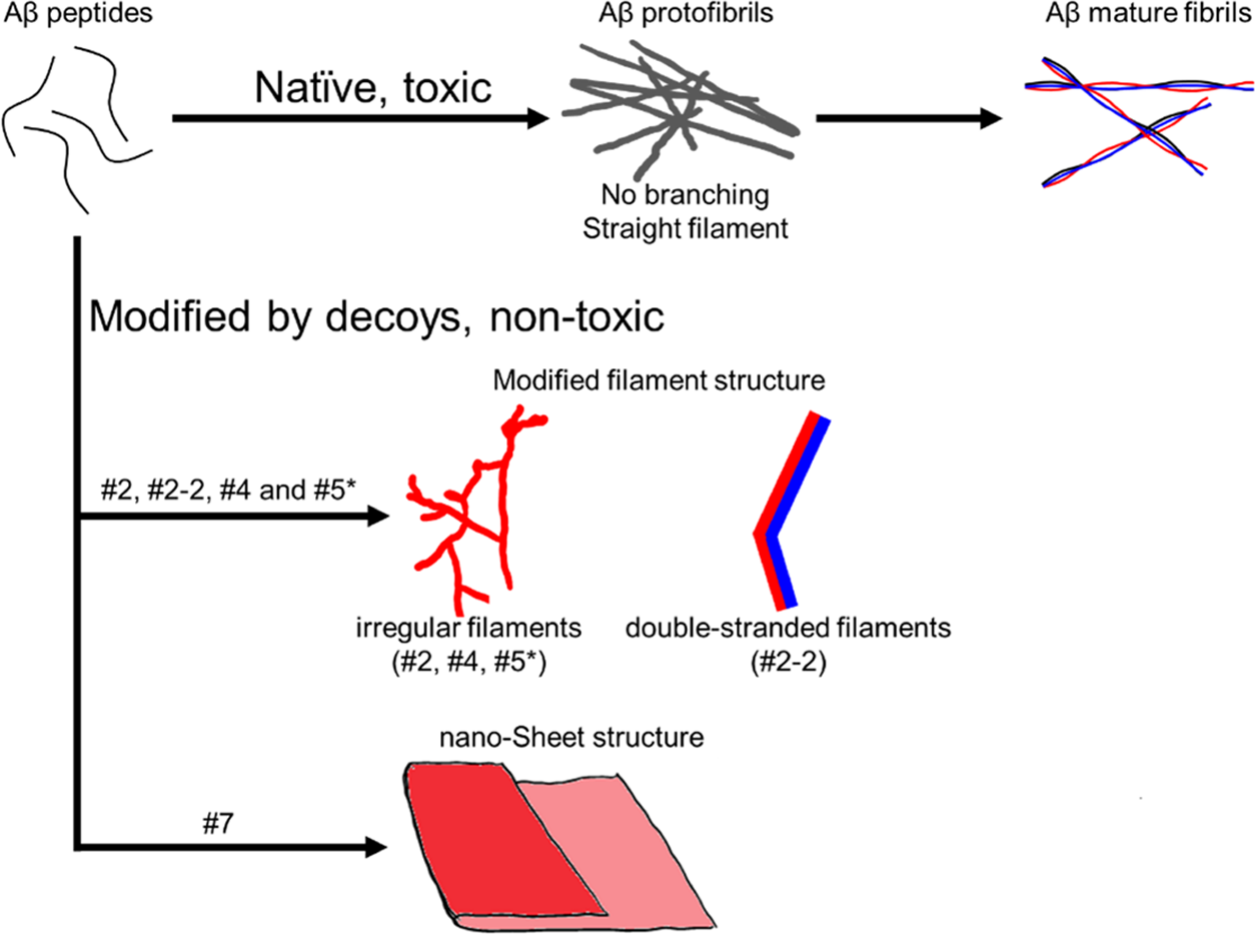
Schematic summary of how small molecular decoys interrupt the “native” Aβ aggregation pathway to protofilament, protofibrils, and finally, fibrils. The soluble aggregates are regarded to be the toxic principle (top row). Several test compounds interrupted the process and prevented fiber formation. Compounds #2, #4, #5 (active in Aβ_42_ only), and #2-2 generated irregular protofilament-like structures (center), whereas compound #7 induced a completely different sheet structure (bottom row). Compound #7 acted as an inducer of aggregation.

As a molecule with 40–42 amino acids, Aβ can be expected to occupy a large number of conformations in solution. This is in common with peptides active as hormones or neurotransmitters. It has been pointed out that in order to adopt a conformation and orientation compatible with signaling, there have to be several segments of contact to account for speed (and accuracy). This suggests a zipper mechanism where each binding step generates a small entropy loss.^25^ In the test tube experiments, this can be described in terms of a lag phase, an exponential growth phase, to an equilibrium phase with protofibrils that can be monitored by ThT labeling.^26^ The current experiments illustrate that this sequence of events can be disturbed by coincubation with substances selected for affinity by pharmacophore search *in silico*. Different compounds disturb these processes differently when bypassing the natural pathway (Figure 8).

Polymorphism in human brain amyloid deposits has also been reported. A principal difference seems to occur between sporadic and familial cases.^27^ Structural heterogeneity and intersubject variability of Aβ in familial and sporadic AD have also been reported.^28^ Indirectly, our data suggest an explanation for amyloid polymorphism.^29^

Other work has indicated that also fibril formation shows structural variation. By characterizing amyloid fibrils in the AD brain specimens with solid-state NMR, Aβ_40_ aggregates were always more common than Aβ_42_ aggregates.^3^ Interestingly, segregating fibril specimens from rapidly (<2 years) progressing AD from cases with long duration (>5 years) revealed characteristic differences, with the rapidly developing variant having more irregularities.^21,22^ Such differences may have consequences for immunotherapy and amyloid-PET imaging. The differences probably arise early during the aggregation process to protofibrils.

### Conclusions

Pharmacophore search among small molecules in a chemical depository identified small molecule candidates for potential inhibition of aggregation of Aβ_40_ and Aβ_42_ into toxic pathways. It is relevant to study both peptides, although there is a consensus that Aβ_42_ is the more toxic. Judging from the present results, most molecules showing activity are active against both peptides and, as observed by fluorescence indicators and TEM imaging, produce similar macrostructures. Compound #5 was a near miss, showing weak activity against Aβ_40_ but full activity against Aβ_42_. This is why we have given this compound extra attention (Figure 6) since it opens a way of higher selectivity, a set goal in drug development.

The active molecules acted as decoys turning the aggregation into nontoxic pathways. The activity was related to a change in the macrostructure of the aggregates observed by TEM. Highly significant were two molecules turning aggregates into a two-dimensional network and double-stranded thin fibrils, respectively. None of the compounds were previously known to be active against Aβ aggregation, illustrating the usefulness of repurposing.^30^

## Materials and Methods

### Pharmacophore Development

For pharmacophore development, we used the interface-based method^9^ and the Pharmacophore Query Editor module of Molecular Operating Environment (MOE) software (CCG, Montreal, Canada). Using this pharmacophore, we conducted docking of 265,242 compounds of the Open NCI Database, release 4 (https://cactus.nci.nih.gov/download/nci/) with 200 conformations per each compound. The details of pharmacophore center elucidation are given in the Supporting Information.

### Chemicals

The structures of tested compounds are shown in Figure S3. The selection of test compounds was based on *in silico* modeling with pharmacophore-based computation (Supporting Information). The chemicals were provided by the repository and used without any further purification. Stock solutions were prepared by dissolving the compounds in 20 mM HEPES buffer (pH 7.4) to 10 mM concentration.

### ThT-FCS Analysis

Human recombinant amyloid-β Aβ_40_ and Aβ_42_ peptides were purchased from AlexoTech AB, Umeå, Sweden, and rPeptide, Georgia, respectively. 50 μg of the peptide was dissolved in 50 μL of 10 mM NaOH and incubated at room temperature for 1 min. The peptide/NaOH solution was diluted to 10 μM peptide concentration with 20 mM HEPES buffer (pH 7.4) with ThT (10 μM final concentration). Test compounds were added to the same molar concentration as the Aβ peptide, and the mixture was stirred thoroughly. The reaction was conducted at room temperature in an air-conditioned room, 21 ± 1 °C, and monitored by ThT-FCS. To this aim, 100 μL of an aliquot was taken at each time point and transferred to an 8-well chambered cover glass (Thermo Fisher Scientific).

CLSM imaging of Aβ precipitates on the cover glass and FCS measurements in the solution were performed using an LSM510 META-ConfoCor3 (Carl Zeiss) instrument for Aβ_40_ or the LSM880 (Carl Zeiss) instrument for Aβ_42_. Both instruments were equipped with a water immersion objective (C-Apochromat, 40×, 1.2 N.A., Corr, Carl Zeiss) and avalanche photodiode detectors (APDs in ConfoCor3) or gallium arsenide phosphide detectors (GaAsPDs in LSM880). ThT was excited using the 458 nm line of the multiline (458, 477, 488, and 514 nm) Ar-ion laser. The pinhole size was 1 airy unit (70 μm in ConfoCor3 and 32 μm in LSM880). The fluorescence signal passed through a bandpass 530–610 filter. For each time point, FCS measurements were carried out in a series of 30 consecutive measurements, each measurement lasting 10 s.

Data acquired by FCS was analyzed by AIM or ZEN software (Carl Zeiss). The average count rate during each 10 s measurement was computed from fluorescence intensity fluctuations. Calculated autocorrelation curves were fitted using a two-component fitting model with a triplet fraction
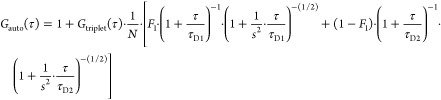
1
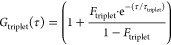
2where *F*_triplet_ is the average fraction in the triplet state, τ_triplet_ is the average relaxation time of the triplet state, τ_D1_ and τ_D2_ are the average diffusion times of the first and second components, respectively, *N* is the average number of fluorescent molecules in the effective observation volume, and *s* is the structure parameter (ratio of long and short radii of the effective observation volume). The effective observation volume was measured in calibration experiments using a rhodamine 6G dye solution (*D*_Rho6G_ = 414 μm^2^/s). Weighted diffusion times of the first and second components were computed to characterize the average size of Aβ aggregates.
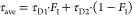
3

### Transmission Electron Microscopy (TEM)

Aβ_40_ or Aβ_42_ peptides were dissolved and diluted to 10 μM peptide concentration, as described above. The test compounds were added to the same molar concentration (10 μM) and the mixture was incubated with stirring for 1 h at room temperature. With test compound #7, Aβ aggregates form rapidly. Aβ solutions with compound #7 were therefore incubated without stirring for 10 min.

Copper 200 mesh EM grids coated with a Formvar/carbon film were hydrophilized in an EMS 100× glow discharge unit (45 s at the current of 25 mA) before use. Five microliters of the freshly prepared sample was applied to the grid and incubated for 1 min at room temperature. The drop was removed with a pipette and the specimen was negatively stained, as previously described.^31^ Briefly, a 5 μL drop of freshly prepared 1% uranyl acetate (UAc) was applied to the grid and incubated for a few seconds. The uranyl drop was then removed with a pipette and a fresh 5 μL UAc drop was applied. The application–removal cycle was repeated seven times, and then the grid was air-dried. The specimens were analyzed using a Talos L120C transmission electron microscope (Thermo Fisher Scientific, Brno, Czech Republic) operating at 120 kV. The images were acquired using a Ceta-D camera.

### Förster Resonance Energy Transfer Coupling with Fluorescence Correlation Spectroscopy (FRET-FCS)

Fluorescently labeled human Aβ_40_ and Aβ_42_, HiLyte Fluor 488-Aβ (Aβ_40,488_ and Aβ_42,488_), and HiLyte Fluor 647-Aβ (Aβ_40,647_ and Aβ_42,647_) were purchased from AnaSpec, Fremont CA. Peptides were dissolved in 5 μL of 10 mM NaOH and diluted with 200 μL of 20 mM HEPES buffer (pH 7.4). Concentrations of the fluorescently labeled peptides were determined by single-color FCS prior to FRET-FCS measurements.

FRET-FCS was performed using the above-described LSM510 META-ConfoCor3 (Carl Zeiss) system for the Aβ_40_ peptide or an LSM780 (Carl Zeiss) microscope system equipped with the same multiline Ar-ion laser and objective lens as the LSM510 system and gallium arsenide phosphide (GaAsP) detectors (500–530 nm for HiLyte 488 and 655–700 nm for HiLyte647) for Aβ_42_ peptides. HiLyte Fluor 488 and was excited using the 488 nm line of the Ar-ion laser. The pinhole size was adjusted to 70 μm in the ConfoCor3 and 40 μm in the LSM780 system. The fluorescence signal was split by NFT 635. The fluorescence signal of HiLyte Fluor 488 and the FRET signal were collected after passing through a bandpass BP505-530 filter (donor channel) and LP655 long pass filter (acceptor channel), respectively. FRET-FCS measurements in the solution were performed in a series of 10 measurements, each measurement lasting 20 s.

Data acquired by FRET-FCS was analyzed by AIM and ZEN software (Carl Zeiss). The autocorrelation curve acquired in the acceptor channel was fitted by a one-component fitting model with a triplet fraction or a two-component fitting model with a triplet fraction for experiments with compound #7. The structure parameter for the green and red channels was determined in calibration experiments using ATTO488 (*D*_ATTO488_ = 400 μm^2^/s) or Cy5 (*D*_Cy5_ = 360 μm^2^/s), respectively. To access interactions between the fluorescently labeled Aβ peptides, molecular brightness, reflected by counts per particle (CPP) in the acceptor channel, was calculated by the ratio of the count rate (CR_R,FRET_) and the number of FRET-positive particles (*N*_R,FRET_).
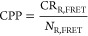
4

### Cell Viability Assay

Human neuroblastoma SH-SY5Y cells were maintained in Dulbecco’s modified Eagle medium (DMEM; Gibco) supplemented with 10% fetal bovine serum (FBS; Gibco) and 1% penicillin and streptomycin (Gibco; final conc. 100 U/mL of penicillin and 100 μg/mL streptomycin).

Solutions of human recombinant Aβ_40_ or Aβ_42_ peptides were prepared as described for ThT-FCS analysis. The SH-SY5Y cells were seeded into an 8-well chambered cover glass (Thermo Fisher Scientific) at a density of (2 × 10^4^ cells/mL × 400 μL) and precultured for 2 days before any treatment. After 2 days of preculturing, the cell culture medium was replaced by fresh FluoroBrite DMEM (Gibco) medium supplemented with 10% FBS and 1% penicillin and streptomycin. Solutions of human recombinant Aβ_40_ or Aβ_42_ without or with the test compound, which were prepared as described for ThT-FCS experiments and allowed to aggregate for 2 days, were added to the cell culture medium in a volume/total volume ratio of 1:3 (equivalent to a final concentration of 3 μM Aβ peptide or 3 μM Aβ peptide with 3 μM compound) and the SH-SY5Y cells were continued to be cultured for 5 days.

To visualize dead cells, the cell-impermeable DNA staining dye 7-AAD (BioLegend, San Diego, CA) was used, and the cell-permeable DNA staining dye Hoechst 33342 (NucBlue Live Ready Probes Reagent; Thermo Fisher Scientific) was used for staining the nuclei. Cells were stained at 37 °C for 30 min and subjected to confocal imaging using the LSM880 (Carl Zeiss) instrument with an objective lens (Plan-Apochromat 20×/0.8 M27, Carl Zeiss) and gallium arsenide phosphide (GaAsP) detectors. Hoechst 33342 and 7-AAD were excited using the 405 and the 543 nm lasers, respectively. The pinhole size was opened as much as possible (600 μm) to maximize fluorescence in the field of view. Fluorescence was detected in the range 410–585 nm (Hoechst 33342) and 548–679 nm (7-AAD). To avoid crosstalk, signals were acquired separately in both channels using the multitrack mode.

The number of whole cells and dead cells in the field of view was counted manually using the ImageJ cell counter plugin. To get adequate statistics on cell viability, we counted at least 2000 cells in each sample. Cell viability (CV) was calculated as
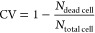
5

### Passive Avoidance (PA) Retention Test *In Vivo*

Thirty-two male C57B1/6J mice from Charles River, 7–8 weeks of age at arrival, were used. The animals were housed in groups of 5 mice in standard cages (A3, 42 × 26 × 20 cm^3^, Macrolon) in a temperature- and humidity-controlled room with a 12 h light/dark cycle (lights on at 6:00 a.m.), with free access to standard lab chow (Ewos R36, Ewos AB, Sodertalje, Sweden) and tap water. The animals were allowed to habituate to the maintenance facilities and were handled by the same experimenter daily for a period of at least five days before the beginning of the experiments. The mice were marked with a pen on the tail during the study. The cages were changed twice a week. Animal housing and experimental procedures followed the protocols and recommendations of the Swedish animal protection legislation. The experimental procedures were approved by the local Animal Ethics Committees (101640) and conformed to the European Council Directive (2010/63/EU). During the experiment, observation of animal health was performed.

Animals were treated with compounds #2, #3, #4, #2-2, and #7 with 5 mg/kg dose and 8 mL/kg injection volume for 5 days by subcutaneous administration (once a day), with the last administration 30–40 min prior to the PA training session (day 1). No compound was given on the test day (24 h after the training session, day 2). The compounds were dissolved with 20 μM HEPES buffer (vehicle). Mice were treated with the vehicle (*n* = 7) and compounds (*n* = 5), respectively.

## Data Availability

Raw data used to generate the figures are available from the corresponding authors, L.T. and S.O., for *in vitro*/*in vivo* experiments and I.F.T. for *in silico* experiments, upon request.

## Supporting Information Available

Ribbon diagram of the annular Aβ_42_ oligomer; Aβ_42_ homodimer interacting residues and distances between them; test compound structures; fluorescence intensity trace in the FCS assay; ThT-responsive Aβ_40_ aggregates precipitated on the coverslip; thickness of Aβ_40_ filaments estimated by TEM imaging; optimization of experimental conditions for FRET-FCS measurements; effects of test compounds on Aβ_42_ peptide aggregation assessed *via* FRET-FCS; characterization of compound toxicity in mice by the PA test (PDF)

## Notes

All fluorescence images and ThT-FCS/FRET-FCS were analyzed by AIM or ZEN software (Carl Zeiss). TEM images were analyzed by ImageJ. The cell viability assay was done on ImageJ with the cell counter plugin. All graphs were made by Origin 2018 software. Statistical analyses were performed using two-sided student’s *t*-test in Microsoft Excel.

## Notes

L.T. and I.F.T. are listed as inventors on a patent related to this work. The authors have no additional financial interests.

## Abbreviations

AD: Alzheimer’s disease
APP: amyloid precursor protein
Aβ: amyloid beta
MD: molecular dynamics
DMEM: Dulbecco’s modified Eagle medium
FBS: fetal bovine serum
FCCS: fluorescence cross-correlation spectroscopy
FCS: fluorescence correlation spectroscopy
FRET: Förster resonance energy transfer
CLSM: confocal laser scanning microscopy
PA: passive avoidance
TEM: transmission electron microscopy
ThT: thioflavin T

## Footnotes

a From the NCI: National Cancer Institute. Downloadable structure files of NCI Open Database Compounds: Release 4.2012. https://cactus.nci.nih.gov/download/nci/ (last accessed 17 Sept, 2022).
