# Supplementary material for: Small Molecule Decoys of Aggregation for Elimination of Aβ-Peptide Toxicity
Source: ACS Chem Neurosci. 2023 Apr 14;14(9):1575–84. doi: 10.1021/acschemneuro.2c00649 (PMC10161222; doi:10.1021/acschemneuro.2c00649)
Supplement: Supplementary file 1 — cn2c00649_si_001.pdf [file cn2c00649_si_001.pdf]

Supplementary Information

Small Molecule Decoys of Aggregation for Elimination of Aβ-Peptide Toxicity

Sho Oasa[a,*], Valentina L. Kouznetsova[b,*], Ann **Tiiman[a], Vladana Vukojević[a], Igor F. Tsigelny[b,c,*], Lars Terenius[a,d,*]**

[a] *Department of Clinical Neuroscience, Center for Molecular Medicine, Karolinska Institutet, SE-171 76, Stockholm, Sweden*

[b] *San Diego Supercomputer Center, University of California San Diego, La Jolla, CA 92093-0505*

[c] *Department of Neurosciences, University of California San Diego, La Jolla, CA 92093-0819*

[d] Corresponding Author: Lars Terenius - *Department of Clinical Neuroscience, Karolinska University Hospital, Karolinska Institutet, SE-17176 Stockholm, Sweden;* Phone: +46 70 330 4985; Email: Lars.Terenius@ki.se

*S.O., V.L.K., I.F.T. and L.T. contributed equally

S1. Annular heptamer of $A\beta_{42}$ based on comprehensive molecular dynamics (MD) simulations[1]

Molecular dynamics simulations were conducted for 100 ns as previously described,[1] using $A\beta_{42}$ NMR structure (PDB ID 1IYT).[2] After consequential docking of seven $A\beta_{42}$ molecules heptamer formation with similar dimeric $A\beta_{42}$ interfaces between monomers was observed (Figure S1).

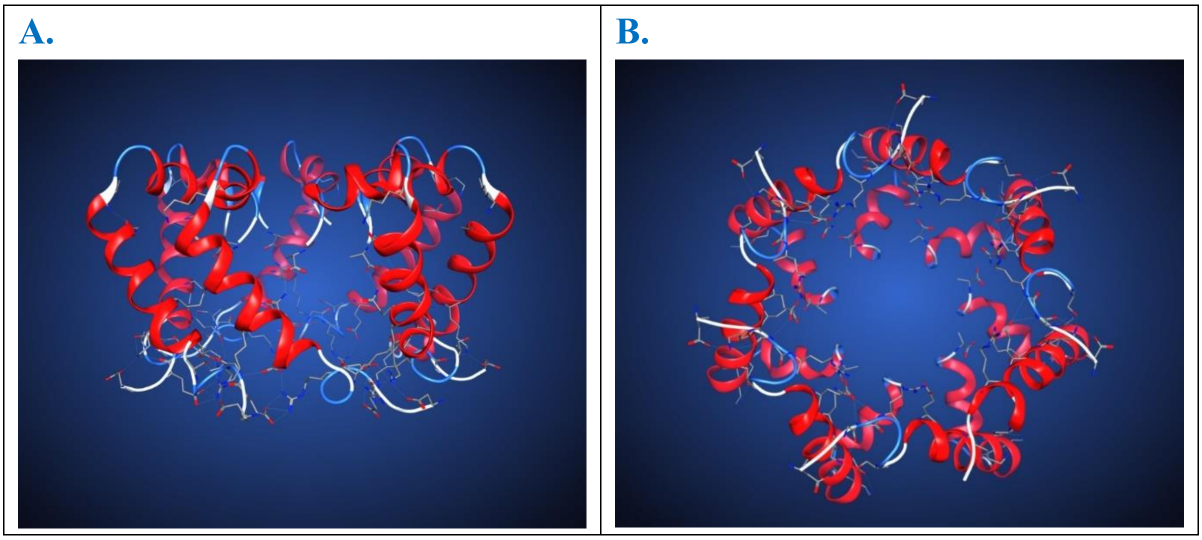

Figure S1. Ribbon diagram of the annular $A\beta_{42}$ oligomer obtained using the most frequently encountered conformation of $A\beta_{42}$ monomer during MD simulations with consistent docking of these conformers to each other. A. *View from the side.* ***B.*** *View from the top.*

S2. Pharmacophore development based on $A\beta_{42}$dimerization interaction

S2.1. Pharmacophore preparation

We analyzed possible intermolecular contacts between two identical molecules of $A\beta_{42}$. A listing of contacts is shown in Table S1.

Table S1. *Aβ42 homodimer interacting residues and distances between them.*

Monomer 1			Monomer 2			Distance, Å	Type
Residue	Atom	Charge	Residue	Atom	Charge		
Arg5	NH2	[N+]	Glu3	OE1	[O]	2.92	H-bond
Val12	CB	[C]	Asp7	CB	[C]	4.30	Hydrophobic
Gln15	CG	[C]	Phe4	CD2	[C]	3.76	Hydrophobic
Lys16	NZ	[N+]	Asp7	OD1	[O]	2.61	H-bond
Lys16	NZ	[N+]	Ser8	OG	[O]	2.67	H-bond
Lys16	CG	[C]	Phe4	CB	[C]	4.44	Hydrophobic
Phe20	ring	[C]	Phe4	CZ	[C]	3.65	Hydrophobic
Phe20	ring	[C]	Tyr10	CE2	[C]	4.27	Hydrophobic

According to Table S1, there are two positive polar and three hydrophobic contacts in Monomer 1 (Figure S2, M1, orange); in Monomer 2 (Figure S2, M2, yellow) there are three negative polar contacts and three hydrophobic contacts. Because Lys16 NZ atom can interact with oxygens of both Asp7 and Ser8, there is only one donating center. Using the Molecular Operating Environment (MOE) Pharmacophore Editor module, we created a final pharmacophore containing two donors: F1:Don and F2:Don, and three hydrophobic centers: F3:Hyd, F4:Hyd, and F5:Hyd (Figure 1).

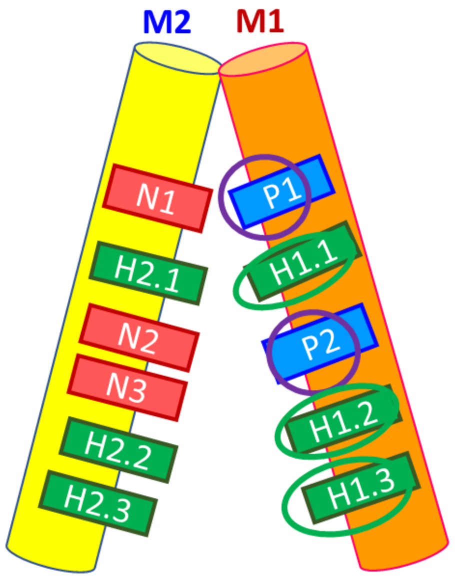

Figure S2. Schematic drawing of the interface between two Aβ42 monomers in the homodimer. *General scheme of residue interactions (red—negative (N), blue—positive (P), green—hydrophobic (H)).*

S2.2. Pharmacophore-based docking

The developed pharmacophore was used in a docking procedure on Open NCI Database, release 4 (May 2012);[1] 265,242 compounds with maximum 200 conformations each. The MOE Compute/Dock module was used. Thirty compounds were docked to Monomer 2 of $A\beta_{42}$ (yellow in Figure S2). The compounds with highest scores were selected for testing.

S3. Test compounds

The test compounds were provided by the National Cancer Institute Chemotherapeutic Agents Repository, Bethesda, MD. They have been identified via in silico modeling of $A\beta_{42}$ dimer formation, which is diverse, and may be able to propagate to oligomerization into fibers or circular forms[1]. Test compounds were dissolved in HEPES buffer (pH 7.4) to yield stock solutions (10 mM concentration) and stored at 4°C.

The first compound structures and numbering (#1-#8) are shown in Figure S3A: 4-amino-3-[[4-[4-[(1-amino-4-sulfonato-2-naphthyl)azo]-2-methyl-phenyl]-3-methyl-phenyl]azo]naphthalene-1-sulfonate (NSC 8676), 4-hydroxy-3-[([4-[4-[(1-hydroxy-4-sulfo-2-naphthyl)azo]-3-methoxy-phenyl]-2-methoxy-phenyl]azo]naphthalene-1-sulfonic acid (NSC 9615), 8-[2-hydroxyethyliminomethyl]-5-isopropyl-3-methyl-2-[1,6,7-trihydroxy-8-[(Z)-2-hydroxyethyliminomethyl]-5-isopropyl-3-methyl-2-naphthyl]naphthalene-1,6,7-triol (NSC 11979), 5-[[4-[[2,4-diamino-5-[(2-methyl-3-sulfo-phenyl)azo]phenyl]azo]phenyl]azo]-2-hydroxy-benzoic acid (NSC 26252), 4-(dimethylamino)-1,6,10,12,12a-pentahydroxy-N-[(3-hydroxypropylamino)methyl]-6-methyl-3,11-dioxo-4,4a,5,5a-tetrahydrotetracene-2-carboxamide;methanesulfonic acid (NSC 69318), [4-[[4-(dimethylamino)-3-sulfo-phenyl]-[4-[methyl-[(3-sulfophenyl)methyl]amino]phenyl]methylene]cyclohexa-2,5-dien-1-ylidene]-dimethyl-ammonium (NSC 75431), N1,N4-bis[3-carbamoyl-5-(4,5-dihydro-1H-imidazol-2-yl)phenyl]terephthalamide (NSC 100873), N1,N4-bis[(4-sulfamoylphenyl)methyl]terephtalamide (NSC 111344).

The second compound structures and numbering (#1-2 - #7-2) are shown in Figure S3B: 2-[3-[(E)-N-[(E)-1-[3-(carboxymethyl)-2,2-dimethyl-cyclobutyl]ethylideneamino]-C-methyl-carbonimidoyl]-2,2-dimethyl-cyclobutyl]acetic acid (NSC 5640), 3-amino-4-[(E)-[4-[4-[(E)-(2-amino-6-sulfonato-1-naphthyl)azo]-3-methyl-phenyl]-2-methyl-phenyl]azo]naphthalene-2,7-disulfonate (NSC 16224), 5-amino-4-hydroxy-3-[(E)-[4-[4-[(E)-(1-hydroxy-4-sulfo-2-naphthyl)azo]-3-methyl-phenyl]-2-methyl-phenyl]azo]naphthalene-2,7-disulfonic acid (NSC 51536), N-[2-chloro-4-[3-chloro-4-(3-oxobutanoylamino)phenyl]phenyl]-3-oxo-butanamide (NSC 165885), 4-[[3-[4-(4,6-diamino-2,2-dimethyl-1,3,5-triazin-1-yl)phenyl]-2-(p-

---

[1] Downloadable structure files of NCI Open Database Compounds: Release 4.2012. https://cactus.nci.nih.gov/download/nci/ (last accessed 17 September 2022).

tolyl)propanoyl]amino]benzenesulfonyl fluoride (NSC 211141), 5-chloro-2-[2-[3-[(3-fluorosulfonyl-4-methyl-phenyl)carbamoylamino]anilino]-2-oxo-ethoxy]benzoic acid (NSC 212044), 4-[6-(3-carboxy-4-hydroxy-1-naphthyl)-6-oxo-hexanoyl]-1-hydroxy-naphthalene-2-carboxylic acid (NSC 292214).

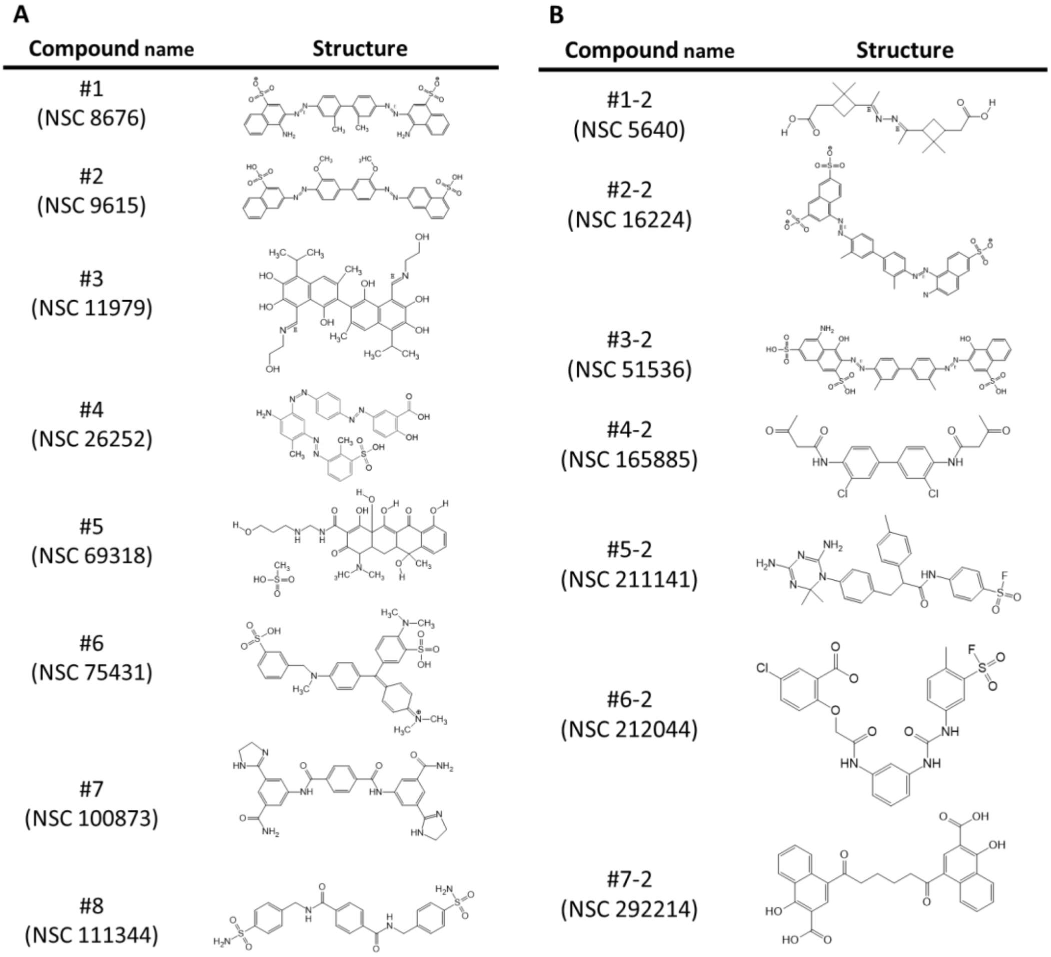

Figure* S3. *Test compound structures. *A. First testing round compounds.* ***B.*** *Second testing round compounds.*

S4. ThT analysis used to screen the activity of test compounds

To quantitatively characterize the effect of test compounds on Aβ_{40} and aggregation, we used Thioflavin T-based Fluorescence Correlation Spectroscopy (ThT-FCS) analysis. In this assay, FCS quantified the fluorescence intensity of ThT and diffusion time (aggregate size) of ThT-responsive Aβ_{40} aggregates in the solution. Most effective inhibitory compounds with regard to their effect on both fluorescent intensity and diffusion time, were different from control without compounds as shown in Figure 2. Compound #5 showed weak activity, causing a delay in the aggregation onset, but eventually leading to the same fluorescence intensity and the same aggregate size (Figure S4).

Compound #1 (NSC 8676) and #6-2 (NSC 212044) showed autofluorescence emission

and precipitation.

The interference of these activities are reasons for exclusion from further testing; it can be noted that apparently a fraction of #1 is interfering with Aβ aggregation (Figure S5C). Compound #6-2 precipitates out of the solution (Figure S6). It is worth pointing out that there is superficial similarity between one of the active structures, compound #2-2, and the histologic dye, Congo Red, which has been studied repeatedly in the past and used as a reference in screening.[3] Congo Red with $A\beta_{40}$ formed precipitate in our protocol. The precipitates were irregular in shape, but with a preference for fibrils and circular forms (data not shown).

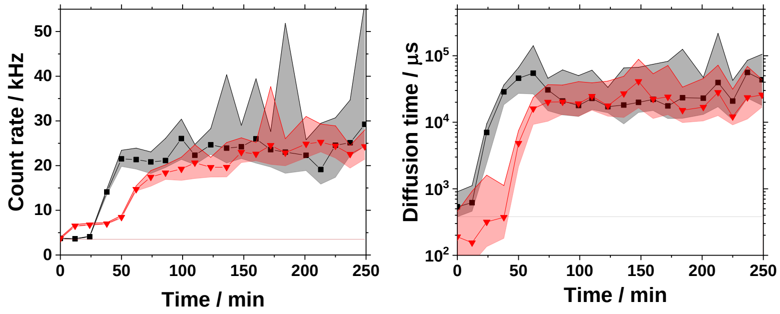

***Figure S4*. Test compound #5 showing weak activity in ThT-FCS analysis.** *Time-lapse ThT-FCS quantified fluorescence intensity of ThT (left) and ThT-positive* $A\beta_{40}$ *aggregate diffusion time (right) in the absence (black) and presence of compound #5 (NSC 69318; red). Plots and regions show median and 25%-75% quartiles of 30 repeats of FCS measurements. This compound is only weakly active with slow onset.*

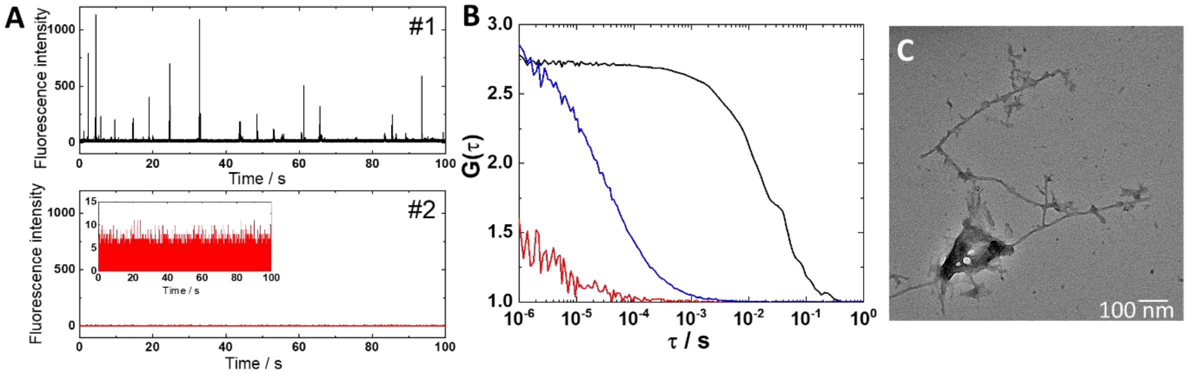

***Figure S5*. Fluorescence intensity trace in FCS assay with compound #1 alone (black) and for comparison compound #2 (red).** *Inset: enlarged fluorescence intensity recording of compound #2. B. Corresponding autocorrelation curves with compound #1 (black) and #2 (red). Blue: autocorrelation curve recorded in ATTO488 solution. C. TEM analysis of Aβ40 aggregates with compound #1.*

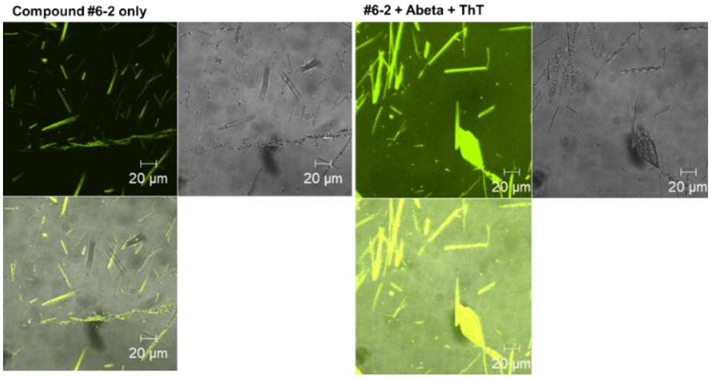

Figure S6. *Fluorescence images of rod-like structures with compound #6-2 alone (left) and compound #6-2 with Aβ40 and ThT (right).*

S5. ThT-positive Aβ40 aggregates precipitated on the coverslip

CLSM imaging of ThT fluorescence revealed that the Aβ40 peptide forms large insoluble aggregates that precipitate on the coverslip (Figure S7). To characterize the fraction on the coverslip, we imaged the coverslip at the end time point in the absence and presence of test compounds. Compounds #4 (NSC 26252) and #2-2 (NSC 16224) showed a smaller number of aggregates than control without compounds. Compound #7 (NSC 100873), on the other hand, precipitated large and differently shaped aggregates. Compound #2 (NSC 9615) precipitated more and smaller aggregates compared to the control. While compound #3 showed no change in aggregation in the liquid phase, intense precipitation of comparatively smaller aggregates was observed on the coverslip. Compound #5 (NSC 69318) showed weak activity in solution (Figure S4), and a smaller number of precipitated aggregates.

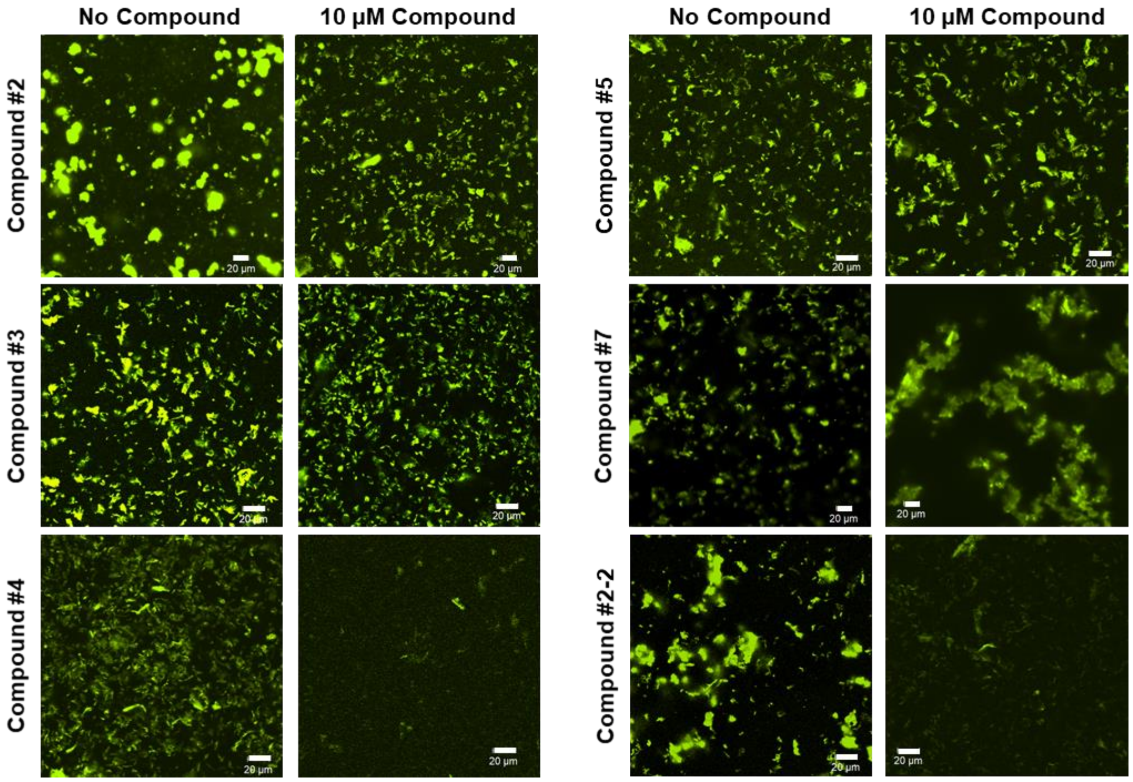

***Figure S7*. ThT-responsive Aβ_{40} aggregates precipitated on the coverslip.** *Fluorescence images of Aβ_{40} precipitates on the coverslip at the end time point of time-lapse ThT-FCS experiments (Figures 2 and S4). Compounds #4 (NSC 26252) and #2-2 (NSC 16224) showed a remarkably small number of aggregates on the coverslip consistent with FCS experiments. Interestingly, compound #7 showed a different precipitate structure on the coverslip. Scale bar: 20 μm.*

S6. Structural characterization of Aβ_{40} precipitates using TEM

Additional trials with compound #7 (NSC 100873) confirmed unique images, different from others (Figure 3), confirming reproducibility of this macrostructure at other positions of same grid (Figures-S8A, S8B) and on another experimental day (Figure S8C).

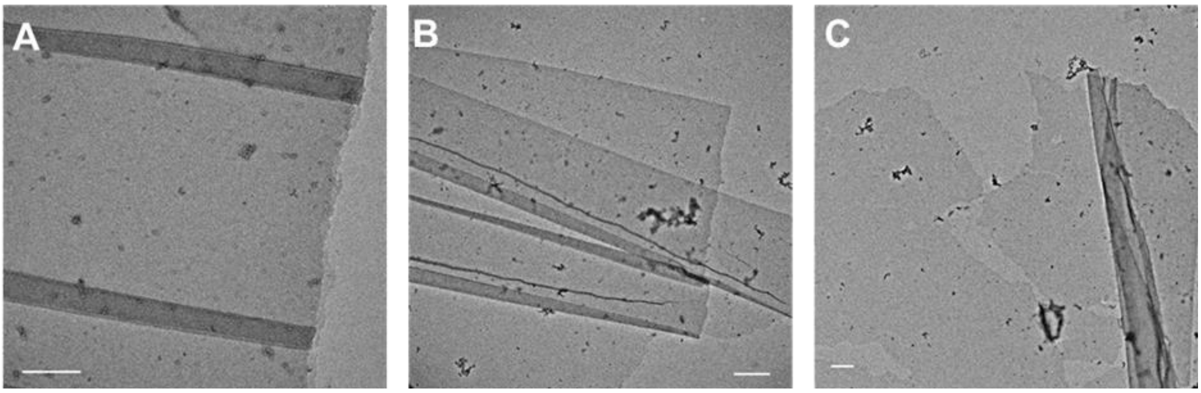

***Figure S8.* Details of Aβ_{40} sheet structure formed in the presence of #7 revealed by TEM imaging. A-C.** *Images acquired at different positions at the same grid (A, B) and on another experimental day (C). Scale bar: 400 nm.*

S7. Thickness of Aβ_{40} filament structures characterized by TEM

We also estimated the thickness of filament structures recorded by TEM images (Figure S9). The control, *i.e.*, no compound sample formed protofibrils (8.0 ± 0.8) nm thick, and mature fibers (15 ± 2.8) nm thick. Compound #5 gave no significant difference in fibril thickness (protofibril: (8.5 ± 0.9) nm; mature fibrils: (15 ± 1) nm). Compounds #2 and #4 formed protofilament-like structures with branching, and with thickness not significantly different from the “no compound” sample (#2: (8.8 ± 0.9) nm; #4: (8.3 ± 1.7) nm). Interestingly, Compound #2-2 formed double-stranded protofilament-like structures as shown in Figure 3. Overall thickness of the double-stranded filament was determined to be similar as Aβ_{40} sample alone (7.5 ± 0.7) nm, constituted by two single filaments of (3.6 ± 0.8) nm, each.

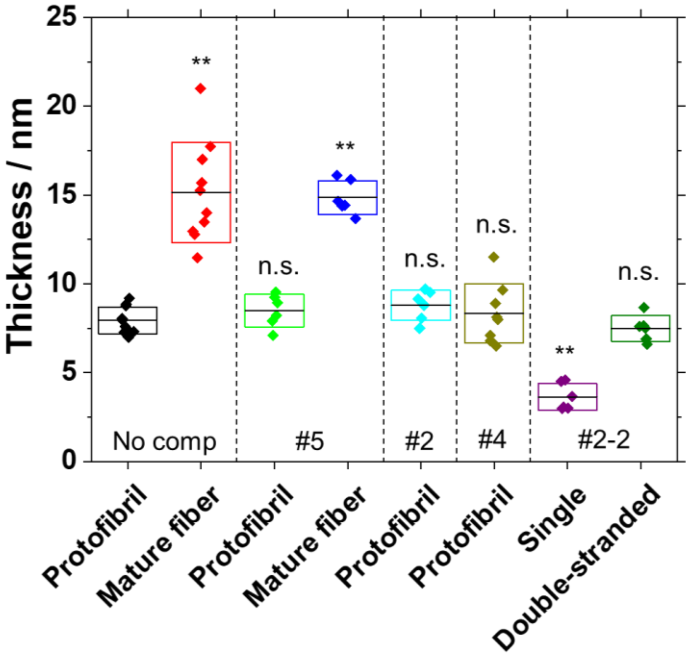

***Figure S9*. Thickness of Aβ$_{40}$ filaments estimated by TEM imaging.** *A t-test was performed to determine if there is a statistically significant difference in the thickness between Aβ$_{40}$ protofilaments formed in the absence (control) and presence of test compound, or between single- versus double-stranded filaments. (** p<.01). No comp = no compound.*

S8. Optimization of FRET-FCS system toward an interaction between spectrally distinct fluorescence-labeled Aβ peptides

FCCS was unable to quantify the interaction of Aβ peptides since no cross-correlation was observed due to large excess of unbound molecules (Figure S10).

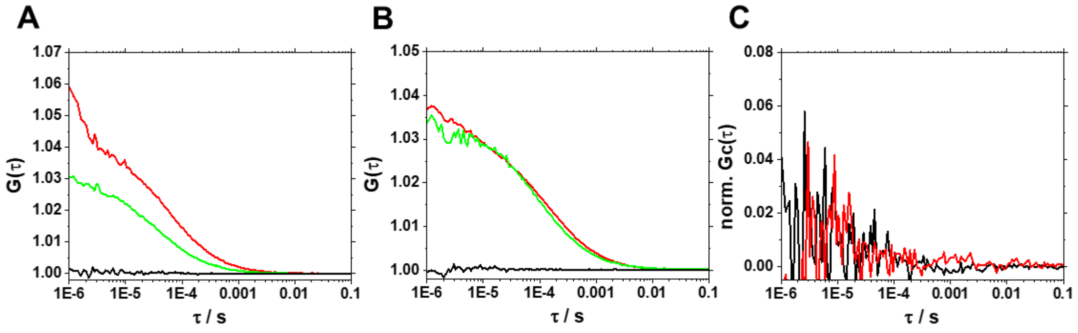

Figure S10*. FCCS measurements on Aβ peptide. A and B.** *Correlation curves of dye mixture (A; negative control) and fluorescently labeled Aβ peptides (B). Green; autocorrelation curve in HiLyte488, red; autocorrelation curve in HiLyte647, black; cross-correlation curve.* ***C. *Cross-correlation curves normalized by an amplitude of autocorrelation curve in HiLyte647. Black; dye mixture, red; Aβ$_{40}$ peptide mixture. No cross-correlation was observed.*

Alternatively, S. Wennmalm and coworkers studied the oligomerization of Aβ peptides labeled by the same fluorescent dyes using FCS integrated with Förster Resonance Energy Transfer (FRET-FCS).[4] To optimize the FRET-FCS system, the same mixture of $A\beta_{40,488}$ and $A\beta_{40,633}$ labeled peptides was subjected to FCCS and FRET-FCS. Although fluorescent bursts are detected in FCCS measurement with red laser, average photon counts level was rifted up to 150 kHz due to all $A\beta_{40,633}$ excited by red laser. On the other hand, switching off the red laser reduced highlighting fluorescent bursts significantly (Figure S11A). These fluorescent bursts were not observed in the single-color peptide solution ($A\beta_{40,488}$ or $A\beta_{40,647}$) (Figure S11C), suggesting that fluorescent bursts originated from the FRET signal of bound peptides. In FRET-FCS the autocorrelation curve showed longer correlation time than in FCCS, detecting only bound components (small oligomers) of Aβ peptides. This suggests that FRET-FCS allows us to assess the interaction of Aβ peptides at an early stage of the aggregation process. Data using this assay are shown in Figures 4, S12, S13.

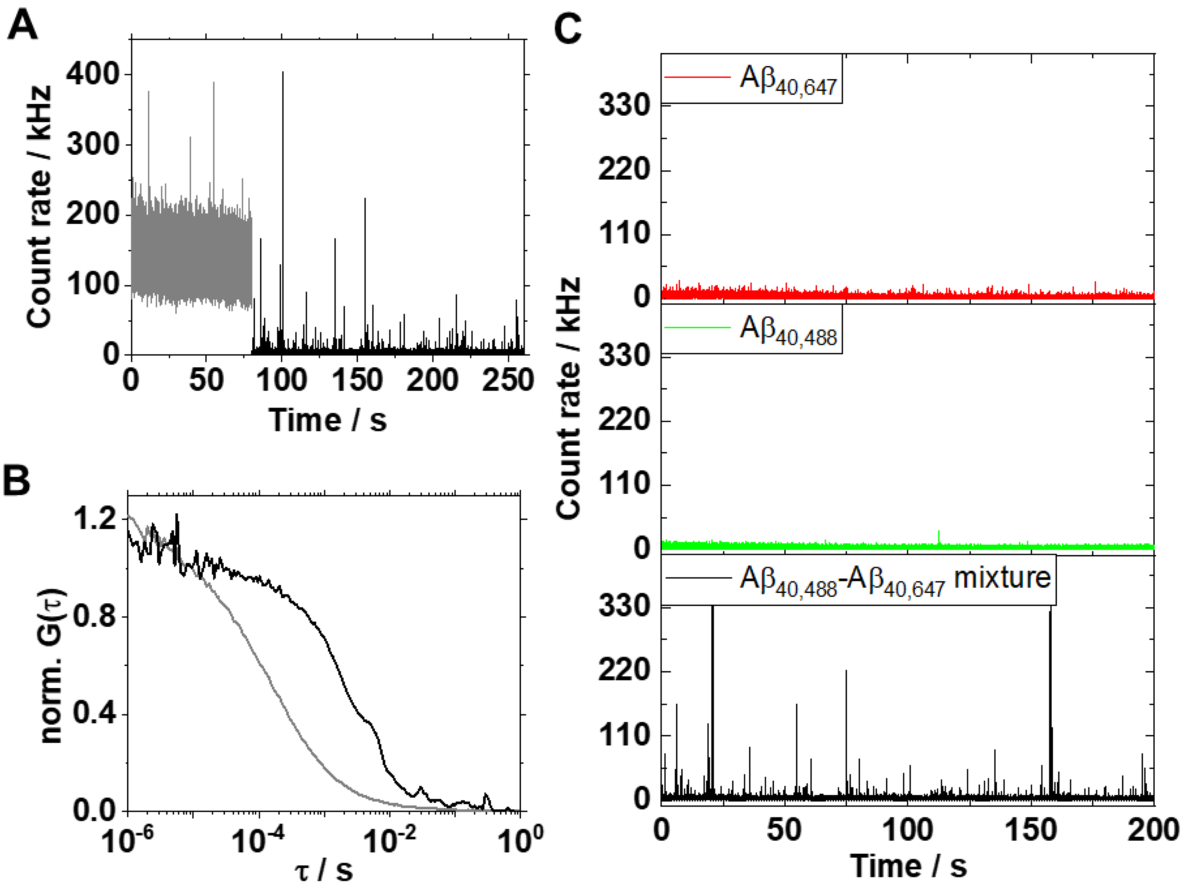

Figure S11. Optimization of experimental conditions for FRET-FCS measurements of $A\beta_{40}$ interactions at an early aggregation stage. A. *Fluorescence intensity trace before (grey) and after (black) switching off the HeNe 633 nm laser while monitoring $A\beta_{40,488}$ and $A\beta_{40,647}$ interactions in the presence of compound #7 (NSC 100873).* ***B.*** *Normalized autocorrelation curves, G(τ) computed from fluorescence intensity fluctuations in Figure S11A before (grey) and after (black) switching off the HeNe 633 nm laser.* ***C.*** *Fluorescence intensity fluctuations*

recorded in a 200 nM solution of A$\beta_{40,647}$ (red), 100 nM A$\beta_{40,488}$ (green) and their mixture when only the 488 nm line of the Ar laser is turned on and the signal is collected using the LP655 long pass emission filter.

S9. Oligomer size of FRET-positive Aβ_{40} peptide complex

To identify the molecular sizes of the FRET-positive complexes, we recorded the diffusion time. Compounds #2, #3, #4 and #2-2 showed similar sized complexes whereas #7 also induced larger complexes, suggesting an inducing effect on aggregation.

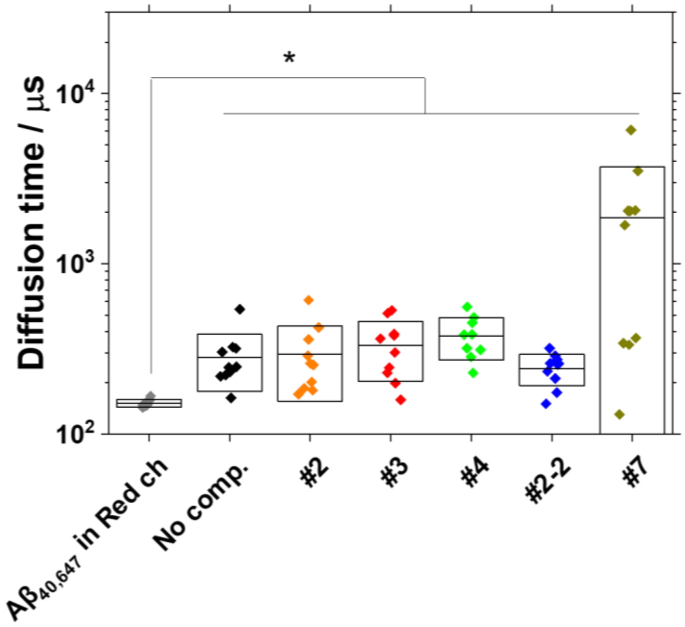

Figure S12.* *Oligomer size of FRET-positive Aβ_{40} peptide complex. *FCS diffusion times in the red channel in the Aβ_{40} peptide mixture (grey: (150 ± 10) µs) and FRET-FCS curves without compound (black: (280 ± 100) µs) and with compounds: #2 (290 ± 140) µs, #3 (330 ± 130) µs, #4 (370 ± 100) µs, #2-2 (250 ± 50) µs, #7 (2 ± 2) ms. Statistical analysis was performed against standard FCS recording in the red channel (grey) (Student t-test, *p<.01).*

S10. Quantitative characterization of compound effect on Aβ42 peptide aggregation by FRET-FCS

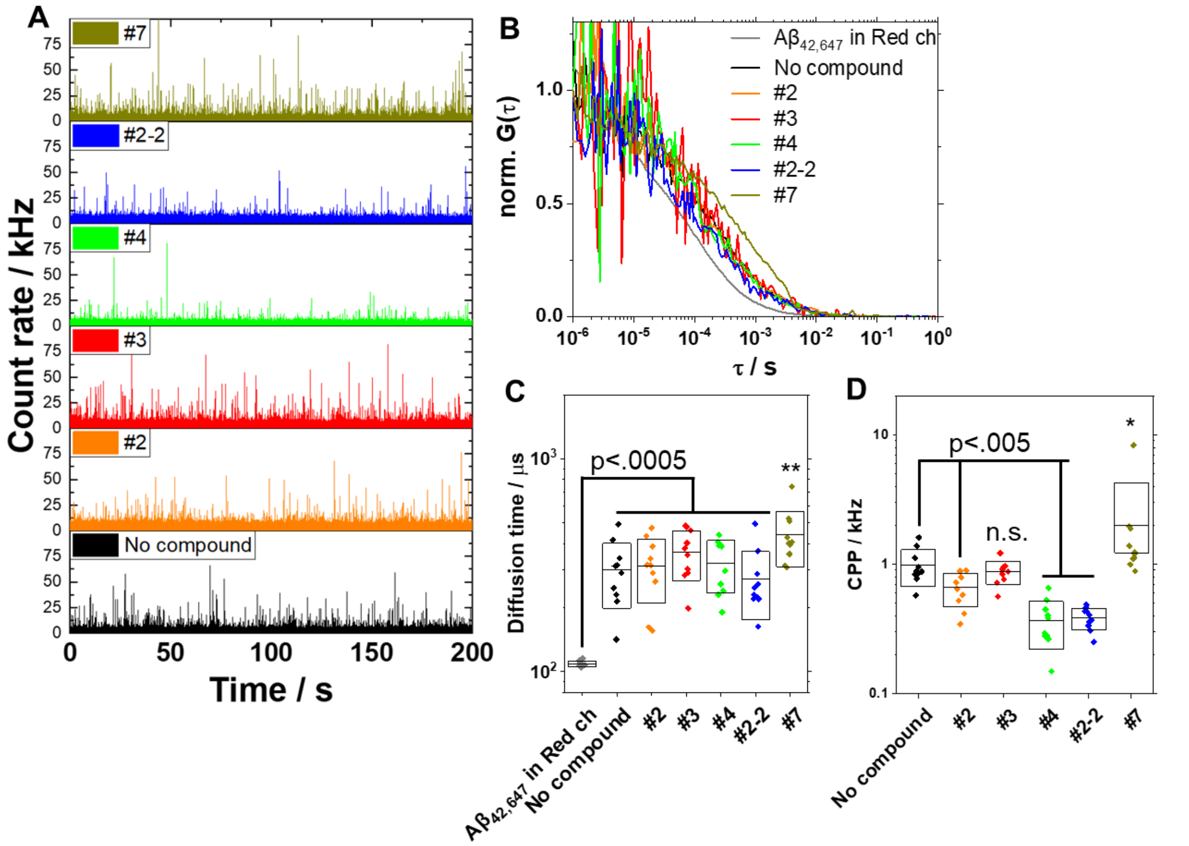

Figure S13. ***Effects of test compounds on Aβ42 peptide aggregation assessed via FRET-FCS.*** *Mixture of 100 nM HiLyte488-Aβ42 (Aβ42,488) and 200 nM HiLyte647-Aβ42 (Aβ42,647) with 300 nM concentration of test compound.* ***A.*** *Photon count level originating through FRET.* ***B.*** *Normalized autocorrelation curve obtained by temporal autocorrelation analysis of fluorescence intensity fluctuations shown in A. The grey curve shows the autocorrelation curve acquired by conventional FCS measurements in an Aβ42,647 solution using the HeNe 633 nm laser to excite HiLyte647.* ***C.*** *Diffusion times expressed as average ± standard deviation. Diffusion time of Aβ42,647 measured by conventional FCS in the red channel, (110 ± 3) µs; diffusion times of FRET-positive Aβ42 oligomers: No compound, (300 ± 100) µs; compound #2, (310 ± 100) µs; #3, (360 ± 100) µs; #4, (320 ± 90) µs; #2-2, (270 ± 100) µs; #7, (440 ± 130) µs. Statistical analysis was performed against conventional FCS recording in the red channel ($p<.0005$) and no compound (**$p<.05$).* ***D.*** *Molecular brightness (CPP; Counts per particle). Average ± Standard deviation: No compound (1.0 ± 0.3), compounds #2 (0.7 ± 0.2), #3 (0.9 ± 0.2), #4 (0.4 ± 0.1), #2-2 (0.4 ± 0.1), #7 (2.0 ± 2.2). Statistical analysis was performed against no compound (**$p<.005$ and *$p<.05$).*

S11. Structural characterization of Aβ42 precipitates using TEM

TEM analysis was also extended to experiments with Aβ42 peptide, using the same protocol as previously used for Aβ40. The recorded macrostructures were identical those previously observed (Figure 3).

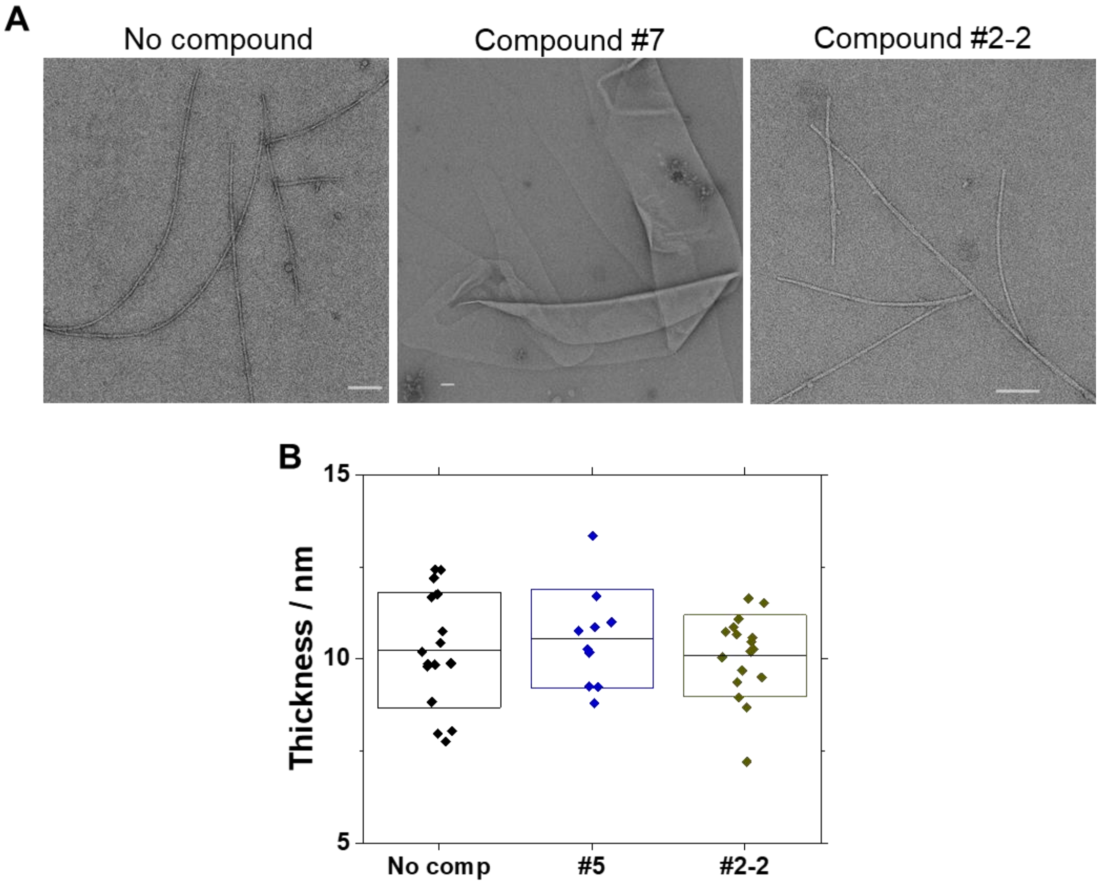

Figure S14. ***Compound effect on the morphology of Aβ42 precipitates. A.*** *Typical TEM images of Aβ42 precipitates without and with test compounds. Aβ42 formed mature filament structure. Aβ42 with compound #7 formed nano-sheet structure similarly as with Aβ40. Compound #2-2 formed branched protofilament structure, partially double-stranded.* ***B.*** *Thickness of Aβ42 filament by TEM image. There are no significant differences in aggregate thickness. Control (No comp), (11 ± 1.5) nm; Compound #5, (11 ± 1.3) nm; and compound #2-2, (10 ± 1.0) nm. Without compound Aβ42 the filament was significantly thinner than mature fibrils of Aβ40, (15 ± 2.8) nm, consistent with the observation by cryo-EM.*[5]

S12. PA test

PA is an associative learning paradigm, based on Pavlovian fear-conditioning[6] and conducted as described earlier.[7] Briefly, a computer-controlled system was used for automatic recording of step-through latencies (Model 256000, TSE system, Bad Homburg, Germany), with two equally sized compartments with a bar floor. The compartments were separated by a 10×10 cm sliding door. The conditioning dark compartment was black and illuminated via indirect light with light intensity of 3 lux. The light compartment was illuminated with a light bulb (24 V, 5 W) with a light intensity of 330 lux. PA training was conducted in a single session on day 1. The animals were treated with the test compounds as described above and after the defined time interval placed in the light compartment with the sliding door closed (i.e., no access to the dark compartment for 60 s). After this delay the sliding door was automatically opened allowing the mouse to access the dark compartment. The latency to enter the dark compartment (training latency) with all four paws was recorded in all animals. Upon entering the dark compartment, the sliding door was automatically closed and a weak electrical current delivered via the grid floor (scrambled current: 2 s duration, 0.4 mA). After exposure to the shock (unconditioned stimulus, UCS), the mouse remained for 30 s in the dark compartment before being transferred to its holding (home) cage to increase the associative strength between the UCS and the training context. Retention latencies were determined on day 2, 24 h after training. The mouse was placed in the light compartment, with access to the dark compartment within 15 s. The latency on day 2 (step-through latency or retention latency) to enter the dark compartment with all four paws was automatically measured with a cut-off time of 300 s.

As shown in Figure S15 the compounds showed no significant activity.

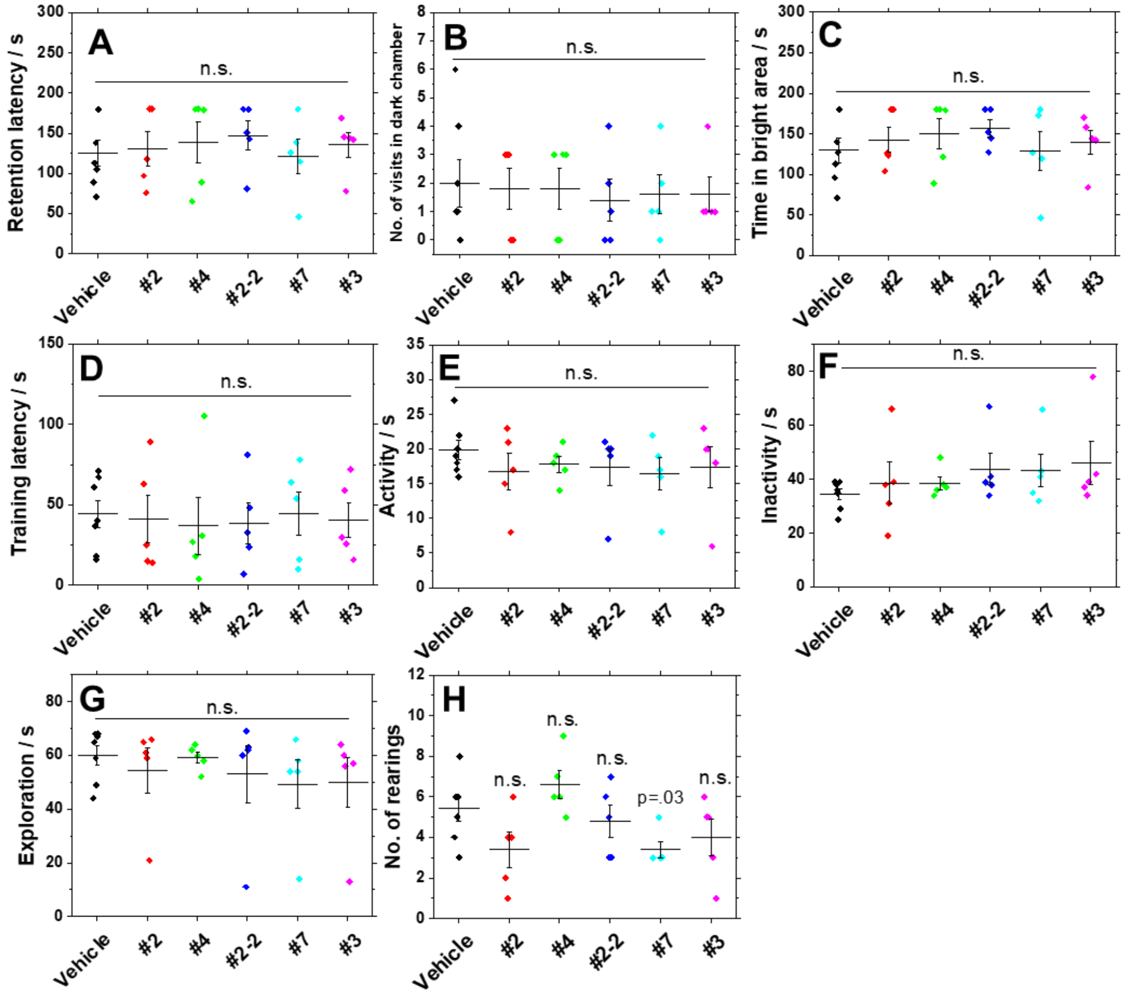

Figure S15. Characterization of compound toxicity in mice by PA test. A-H. *Passive Avoidance (PA) test with compounds injected subcutaneously (5 mg/kg) into mice. Number of mice was 7 (for vehicle 20 µM HEPES buffer) and 5 (each compound in vehicle). Statistical analysis was performed against vehicle.* ***A.*** *Retention latency,* ***B.*** *Number of visits in dark chamber,* ***C.*** *Time in bright area,* ***D.*** *Training latency,* ***E.*** *Activity (duration of locomotor activity more than 5 cm/s),* ***F.*** *Inactivity (duration of activity less than 5 cm/s),* ***G.*** *Exploration (forward locomotor activity in which two photon beams one after the other are broken),* ***H.*** *Rearings (rising and standing only on hind legs).*

References

1. Tsigelny, I. F.; Sharikov, Y.; Kouznetsova, V. L.; Greenberg, J. P.; Wrasidlo, W.; Gonzalez, T.; Desplats, P.; Michael, S. E.; Trejo-Morales, M.; Overk, C. R.; Masliah, E., Structural diversity of Alzheimer's disease amyloid-beta dimers and their role in oligomerization and fibril formation. *J Alzheimers Dis* **2014,** *39* (3), 583-600.
2. Crescenzi, O.; Tomaselli, S.; Guerrini, R.; Salvadori, S.; D'Ursi, A. M.; Temussi, P. A.; Picone, D., Solution structure of the Alzheimer amyloid beta-peptide (1-42) in an apolar

microenvironment. Similarity with a virus fusion domain. *Eur J Biochem* **2002,** *269* (22), 5642-8.
3. Young, L. M.; Saunders, J. C.; Mahood, R. A.; Revill, C. H.; Foster, R. J.; Tu, L. H.; Raleigh, D. P.; Radford, S. E.; Ashcroft, A. E., Screening and classifying small-molecule inhibitors of amyloid formation using ion mobility spectrometry-mass spectrometry. *Nat Chem* **2015,** *7* (1), 73-81.
4. Wennmalm, S.; Chmyrov, V.; Widengren, J.; Tjernberg, L., Highly Sensitive FRET-FCS Detects Amyloid beta-Peptide Oligomers in Solution at Physiological Concentrations. *Anal Chem* **2015,** *87* (23), 11700-5.
5. Schmidt, M.; Rohou, A.; Lasker, K.; Yadav, J. K.; Schiene-Fischer, C.; Fandrich, M.; Grigorieff, N., Peptide dimer structure in an Abeta(1-42) fibril visualized with cryo-EM. *Proc Natl Acad Sci U S A* **2015,** *112* (38), 11858-63.
6. Baarendse, P. J.; van Grootheest, G.; Jansen, R. F.; Pieneman, A. W.; Ogren, S. O.; Verhage, M.; Stiedl, O., Differential involvement of the dorsal hippocampus in passive avoidance in C57bl/6J and DBA/2J mice. *Hippocampus* **2008,** *18* (1), 11-9.
7. Madjid, N.; Tottie, E. E.; Lüttgen, M.; Meister, B.; Sandin, J.; Kuzmin, A.; Stiedl, O.; Ogren, S. O., 5-Hydroxytryptamine 1A receptor blockade facilitates aversive learning in mice: interactions with cholinergic and glutamatergic mechanisms. *J Pharmacol Exp Ther* **2006,** *316* (2), 581-91.
